# Analysis of Known Linear Distributed Average Consensus Algorithms on Cycles and Paths

**DOI:** 10.3390/s18040968

**Published:** 2018-03-24

**Authors:** Jesús Gutiérrez-Gutiérrez, Marta Zárraga-Rodríguez, Xabier Insausti

**Affiliations:** Department of Biomedical Engineering and Sciences, Tecnun, University of Navarra, Manuel Lardizábal 13, 20018 San Sebastián, Spain; mzarraga@tecnun.es (M.Z.-R.); xinsausti@tecnun.es (X.I.)

**Keywords:** average consensus algorithms, distributed computation, sensor networks, convergence time, number of transmissions

## Abstract

In this paper, we compare six known linear distributed average consensus algorithms on a sensor network in terms of convergence time (and therefore, in terms of the number of transmissions required). The selected network topologies for the analysis (comparison) are the cycle and the path. Specifically, in the present paper, we compute closed-form expressions for the convergence time of four known deterministic algorithms and closed-form bounds for the convergence time of two known randomized algorithms on cycles and paths. Moreover, we also compute a closed-form expression for the convergence time of the fastest deterministic algorithm considered on grids.

## 1. Introduction

A distributed averaging (or average consensus) algorithm obtains in each sensor the average (arithmetic mean) of the values measured by all the sensors of a sensor network in a distributed way.

The most common distributed averaging algorithms are linear and iterative:

(1) x(t+1)=W(t)x(t),t∈{0,1,2,…},

where:

(2) $$x\left(t\right)=\left(\begin{array}{c} x_{1}\left(t\right)\\ \vdots \\ x_{n}\left(t\right) \end{array}\right)$$

is a real vector, *n* is the number of sensors of the network, which we label $v_{j}$ with j∈{1,…,n} , $x_{j}\left(0\right)$ is the value measured by the sensor $v_{j}$ , $x_{j}\left(t\right)$ is the value computed by the sensor $v_{j}$ in time t≠0 and the weighting matrix W(t) is an n×n real sparse matrix satisfying that if two sensors $v_{j}$ and $v_{k}$ are not connected (i.e., if $v_{j}$ and $v_{k}$ cannot interchange information), then ${\left[W\left(t\right)\right]}_{j,k}=0$ . From the point of view of communication protocols, there exist efficient ways of implementing synchronous algorithms of the form of (1). (see, e.g., [1]). The linear distributed averaging algorithms can be classified as deterministic or randomized depending on the nature of the weighting matrices W(t) .

### 1.1. Deterministic Linear Distributed Averaging Algorithms

Several well-known deterministic linear distributed averaging algorithms can be found in [2] and [3]. Those algorithms are time-invariant and have symmetric weights, that is, the deterministic weighting matrix W(t) is symmetric and does not depend on *t* (and consequently, $x\left(t\right)=W^{t}x\left(0\right)$ ).

In [2], the authors search among all the symmetric weighting matrices *W* the one that makes (1) the fastest possible and show that such a matrix can be obtained by numerically solving a convex optimization problem. This algorithm is called the fastest linear time-invariant (LTI) distributed averaging algorithm for symmetric weights. It should be mentioned that in [4], the authors proposed an in-network algorithm for finding such an optimal weighting matrix.

In [2], the authors also give a slower algorithm: the fastest constant edge weights algorithm. In this other algorithm, they consider a particular structure of symmetric weighting matrices that depends on a single parameter and find the value of that parameter that makes (1) the fastest possible.

In [3], another two algorithms can be found: the maximum-degree weights algorithm and the Metropolis–Hastings algorithm.

For other deterministic linear distributed averaging algorithms, we refer the reader to [5] and the references therein.

### 1.2. Randomized Linear Distributed Averaging Algorithms

For the randomized case, a well-known linear distributed averaging algorithm was given in [6]. That algorithm is called the pairwise gossip algorithm because only two randomly-selected sensors interchange information at each time instant *t*.

Another well-known randomized algorithm can be found in [7]. That algorithm is called the broadcast gossip algorithm because a single sensor is randomly selected at each time instant *t* and broadcasts its value to all its neighboring sensors. The broadcast gossip algorithm is a linear distributed consensus algorithm rather than a linear distributed averaging algorithm. However, the broadcast gossip algorithm converges to a random consensus value, which is, in expectation, the average of the values measured by all the sensors of the network. If one uses the directed version of the broadcast gossip algorithm [8] in a symmetric graph, one would converge to the true average.

For other randomized linear distributed averaging algorithms, we refer the reader to [9] and the references therein. The linear distributed averaging algorithms reviewed in Section 1.1 and Section 1.2 are the most cited algorithms in the literature on the topic.

### 1.3. Our Contribution

A key feature of a distributed averaging algorithm is its convergence time, because it allows one to establish the stopping criterion for the iterative algorithm. The convergence time is defined as the number of iterations *t* required in (1) until the effective value computed by the sensors, x(t) , has approached the steady state sufficiently close (to a threshold ϵ ). In the literature, we have not found closed-form expressions for the convergence time of the six linear distributed averaging algorithms mentioned in Section 1.1 and Section 1.2. A mathematical expression is said to be a closed-form expression if it is written in terms of a finite number of elementary functions (i.e., in terms of a finite number of constants, arithmetic operations, roots, exponentials, natural logarithms and trigonometric functions). In the present paper, we compute closed-form expressions for the convergence time of the deterministic algorithms and closed-form upper bounds for the convergence time of the randomized algorithms on two common network topologies: the cycle and the path. Observe that these closed-form formulas give us upper bounds for the convergence time of the considered algorithms (stopping criteria) on any network that contains as a subgraph a cycle or a path with the same number of sensors. Specifically, in this paper, we compute:

- a closed-form expression for the convergence time of the fastest LTI distributed averaging algorithm for symmetric weights on the considered topologies (see Section 2.1); moreover, we also compute a closed-form expression for the convergence time of this algorithm on a grid;
- a closed-form expression for the convergence time of the fastest constant edge weights algorithm on the considered topologies (see Section 2.2);
- a closed-form expression for the convergence time of the maximum-degree weights algorithm on the considered topologies (see Section 2.3);
- a closed-form expression for the convergence time of the Metropolis–Hastings algorithm on the considered topologies (see Section 2.3);
- closed-form lower and upper bounds for the convergence time of the pairwise gossip algorithm on the considered topologies (see Section 3.1);
- closed-form lower and upper bounds for the convergence time of the broadcast gossip algorithm on the considered topologies (see Section 3.2).

From these closed-form formulas, we study the asymptotic behavior of the convergence time of the considered algorithms as the number of sensors of the network grows. The obtained asymptotic and non-asymptotic results allow us to compare the considered algorithms in terms of convergence time and, consequently, in terms of the number of transmissions required, as well (see Section 4 and Section 5). The knowledge of the number of transmissions required lets us know the energy consumption of the distributed technique. The knowledge of the energy consumption is a key factor in the design of a new wireless sensor network (WSN), where one has to decide the number of nodes and the network topology. It should be mentioned that when designing new WSNs, cycles, paths and grids are topologies that are considered frequently.

## 2. Convergence Time of Deterministic Linear Distributed Averaging Algorithms

Different definitions of convergence time are used in the literature. We have found three different definitions for the convergence time of a deterministic linear distributed averaging algorithm (see [2,10,11]). In this paper, we consider the definition of ϵ -convergence time given in [11]:

(3) $$\tau \left(\epsilon ,{\left\{W\left(t\right)\right\}}_{t\geq 0}\right):=\min\left\{t_{0}:\frac{∥x\left(t\right)-P_{n}{x\left(0\right)∥}_{2}}{∥x\left(0\right)-P_{n}{x\left(0\right)∥}_{2}}\leq \epsilon ,\forall t\geq t_{0},\forall x\left(0\right)\neq P_{n}x\left(0\right)\right\},$$

where ϵ∈(0,1) , ${∥\cdot ∥}_{2}$ is the spectral norm and $P_{n}:=\frac{1}{n}1_{n}1_{n}^{⊤}$ , with $1_{n}$ being the n×1 matrix of ones and *⊤* denoting the transpose. If we replace the spectral norm by the infinity norm in that definition, we obtain the definition of ϵ -convergence time given in [10]. If the deterministic matrix W(t) in (1) does not depend on *t*, we denote the ϵ -convergence time by τ(ϵ,W) .

### 2.1. Convergence Time of the Fastest LTI Distributed Averaging Algorithm for Symmetric Weights

**Figure 1:**
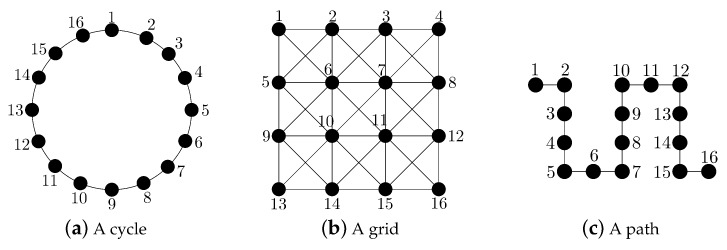
Considered network topologies with 16 sensors.

In this section, we give a closed-form expression for the ϵ -convergence time of the fastest LTI distributed averaging algorithm for symmetric weights, and we study its asymptotic behavior as the number of sensors of the network grows. We consider three common network topologies: the cycle, the grid and the path (see Figure 1).

#### 2.1.1. The Cycle

Let:

(4) $${\overset{\circ }{W}}_{n}\left(\gamma \right):=\;\;\left(\;\;\;\begin{array}{ccccccc} 1\;\;-\;\;2\gamma & \gamma & 0 & \cdots & 0 & 0 & \gamma \\ \gamma & 1\;\;-\;\;2\gamma & \gamma & \cdots & 0 & 0 & 0\\ 0 & \gamma & 1\;\;-\;\;2\gamma & \cdots & 0 & 0 & 0\\ \vdots & \ddots & \ddots & \ddots & \ddots & \vdots & \vdots \\ 0 & 0 & 0 & \cdots & 1\;\;-\;\;2\gamma & \gamma & 0\\ 0 & 0 & 0 & \cdots & \gamma & 1\;\;-\;\;2\gamma & \gamma \\ \gamma & 0 & 0 & \cdots & 0 & \gamma & 1\;\;-\;\;2\gamma \end{array}\;\;\;\right).$$

Using (4), Theorem 1 gives the expression of the weighting matrix of the fastest LTI distributed averaging algorithm for symmetric weights on a cycle with *n* sensors.

**Theorem** **1.** *Let n∈N , with n>3 . Then, ${\overset{\circ }{W}}_{n}\left(\gamma_{0}\right)$ is the weighting matrix of the fastest LTI distributed averaging algorithm for symmetric weights on a cycle with n sensors, where:*

(5) $$\gamma_{0}=\frac{1}{2-\cos\frac{2\pi }{n}-\cos\frac{2\pi (j_{0}-1)}{n}},$$

*with:*

(6) $$\begin{array}{c} j_{0}=\left\{\begin{array}{cc} \frac{n}{2}+1 & \mathrm{if}\;n\;\mathrm{is}\;\mathrm{even},\\ \frac{n+1}{2} & \mathrm{if}\;n\;\mathrm{is}\;\mathrm{odd}. \end{array}\right. \end{array}$$

**Proof.**  See Appendix B.  ☐

We now give a closed-form expression for the ϵ -convergence time of the fastest LTI distributed averaging algorithm for symmetric weights on a cycle. We also study the asymptotic behavior of this convergence time as the number of sensors of the cycle grows.

We first introduce some notation: Two sequences of numbers $\left\{a_{n}\right\}$ and $\left\{b_{n}\right\}$ are said to be asymptotically equal, and write $a_{n}∼b_{n}$ , if and only if $\lim_{n\to \infty }\frac{a_{n}}{b_{n}}=1$ (see, e.g., [12] (p. 396)), and, consequently,

(7) $$\tau \left(\epsilon ,{\overset{\circ }{W}}_{n}\left(\gamma_{0}\right)\right)=\Theta \left(n^{2}\log\epsilon^{-1}\right).$$

Let f,g:N→R be two non-negative functions. We write f(n)=O(g(n)) (respectively, f(n)=Ω(g(n)) ) if there exist K∈(0,∞) and $n_{0}\in N$ such that f(n)≤Kg(n) (respectively, f(n)≥Kg(n) ) for all $n\geq n_{0}$ . If f(n)=O(g(n)) and f(n)=Ω(g(n)) , then we write f(n)=Θ(g(n)) .

**Theorem** **2.** *Consider ϵ∈(0,1) and n∈N , with n>3 . Let ${\overset{\circ }{W}}_{n}\left(\gamma_{0}\right)$ be as in Theorem 1. Then,*

(8) $$\tau \left(\epsilon ,{\overset{\circ }{W}}_{n}\left(\gamma_{0}\right)\right)=\left\{\begin{array}{cc} \left\lceil \frac{\log\epsilon^{-1}}{-\log\frac{1+\cos\frac{2\pi }{n}}{3-\cos\frac{2\pi }{n}}}\right\rceil & \mathrm{if}\;n\;\mathrm{is}\;\mathrm{even},\;\\ \left\lceil \frac{\log\epsilon^{-1}}{-\log\frac{\cos\frac{\pi }{n}+\cos\frac{2\pi }{n}}{2+\cos\frac{\pi }{n}-\cos\frac{2\pi }{n}}}\right\rceil & \mathrm{if}\;n\;\mathrm{is}\;\mathrm{odd}, \end{array}\right.$$

*where log is the natural logarithm and ⌈x⌉ denotes the smallest integer not less than x. Moreover,*

(9) $$\tau \left(\epsilon ,{\overset{\circ }{W}}_{n}\left(\gamma_{0}\right)\right)∼\frac{n^{2}\log\epsilon^{-1}}{2\pi^{2}},$$

**Proof.**  See Appendix C.  ☐

Since the number of transmissions per iteration on a cycle with *n* sensors is *n* for the fastest LTI distributed averaging algorithm for symmetric weights, the total number of transmissions required for $\tau (\epsilon ,{\overset{\circ }{W}}_{n}\left(\gamma_{0}\right))$ iterations is $T\left(\epsilon ,{\overset{\circ }{W}}_{n}\left(\gamma_{0}\right)\right):=n\tau \left(\epsilon ,{\overset{\circ }{W}}_{n}\left(\gamma_{0}\right)\right)$ . From Theorem 2, we obtain:

(10) $$T\left(\epsilon ,{\overset{\circ }{W}}_{n}\left(\gamma_{0}\right)\right)∼\frac{n^{3}\log\epsilon^{-1}}{2\pi^{2}},$$

and hence, $T\left(\epsilon ,{\overset{\circ }{W}}_{n}\left(\gamma_{0}\right)\right)=\Theta \left(n^{3}\log\epsilon^{-1}\right)$ .

#### 2.1.2. The Grid

Let:

(11) $${\tilde{W}}_{n}\left(\alpha \right):=\left(\begin{array}{ccccc} 1-\alpha & \alpha & & & \\ \alpha & 1-2\alpha & \alpha & & \\ & \ddots & \ddots & \ddots & \\ & & \alpha & 1-2\alpha & \alpha \\ & & & \alpha & 1-\alpha \end{array}\right)$$

be the n×n matrix for n≥2 , and ${\tilde{W}}_{1}\left(\alpha \right):=1$ . We define:

(12) $${\overset{⊠}{W}}_{r,c}\left(\alpha \right):={\tilde{W}}_{r}\left(\alpha \right)⊗{\tilde{W}}_{c}\left(\alpha \right),$$

where ⊗ is the Kronecker product. Using (12), Theorem 3 gives the expression of the weighting matrix of the fastest LTI distributed averaging algorithm for symmetric weights on a grid of *r* rows and *c* columns.

**Theorem** **3.** *Let r,c∈N , with rc>2 . Then, the rc×rc matrix ${\overset{⊠}{W}}_{r,c}\left(\frac{1}{2}\right)$ is the weighting matrix of the fastest LTI distributed averaging algorithm for symmetric weights on a grid of r rows and c columns.*

**Proof.**  See Appendix D.  ☐

We now give a closed-form expression for the ϵ -convergence time of the fastest LTI distributed averaging algorithm for symmetric weights on a grid of *r* rows and *c* columns. We also study the asymptotic behavior of this convergence time as the number of rows of the grid grows.

**Theorem** **4.** *Consider ϵ∈(0,1) and r,c∈N , with rc>2 . Without loss of generality, we assume r≥c . Then,*

(13) $$\tau \left(\epsilon ,{\overset{⊠}{W}}_{r,c}\left(\frac{1}{2}\right)\right)=\left\lceil \frac{\log\epsilon^{-1}}{-\log\cos\frac{\pi }{r}}\right\rceil .$$

*Moreover,*

(14) $$\tau \left(\epsilon ,{\overset{⊠}{W}}_{r,c}\left(\frac{1}{2}\right)\right)∼\frac{2r^{2}\log\epsilon^{-1}}{\pi^{2}}$$

*and consequently,*

(15) $$\tau \left(\epsilon ,{\overset{⊠}{W}}_{r,c}\left(\frac{1}{2}\right)\right)=\Theta \left(r^{2}\log\epsilon^{-1}\right).$$

**Proof.**  From [2] (Theorem 1), Theorem A1 and (A64), we obtain (13). The rest of the proof runs as the proof of Theorem 2.  ☐

Since the number of transmissions per iteration on a grid of *r* rows and *c* columns is rc for the fastest LTI distributed averaging algorithm for symmetric weights, the total number of transmissions required for $\tau \left(\epsilon ,{\overset{⊠}{W}}_{r,c}\left(\frac{1}{2}\right)\right)$ iterations is:

(16) $$T\left(\epsilon ,{\overset{⊠}{W}}_{r,c}\left(\frac{1}{2}\right)\right):=rc\tau \left(\epsilon ,{\overset{⊠}{W}}_{r,c}\left(\frac{1}{2}\right)\right).$$

If $r=c=\sqrt{n}$ , from Theorem 4, we obtain:

(17) $$T\left(\epsilon ,{\overset{⊠}{W}}_{r,c}\left(\frac{1}{2}\right)\right)∼\frac{2n^{2}\log\epsilon^{-1}}{\pi^{2}},$$

and hence, $T\left(\epsilon ,{\overset{⊠}{W}}_{r,c}\left(\frac{1}{2}\right)\right)=\Theta \left(n^{2}\log\epsilon^{-1}\right)$ . Observe that from (13), the optimal configuration for a grid with *n* sensors is obtained when $r=c=\sqrt{n}$ .

#### 2.1.3. The Path

Since the path with *n* sensors can be seen as a grid of *n* rows and one column, from Theorem 3, we conclude that ${\tilde{W}}_{n}\left(\frac{1}{2}\right)$ is the weighting matrix of the fastest LTI distributed averaging algorithm for symmetric weights on a path of *n* sensors, and from Theorem 4, we conclude that:

(18) $$\tau \left(\epsilon ,{\tilde{W}}_{n}\left(\frac{1}{2}\right)\right)=\left\lceil \frac{\log\epsilon^{-1}}{-\log\cos\frac{\pi }{n}}\right\rceil .$$

Moreover,

(19) $$\tau \left(\epsilon ,{\tilde{W}}_{n}\left(\frac{1}{2}\right)\right)∼\frac{2n^{2}\log\epsilon^{-1}}{\pi^{2}}$$

and consequently,

(20) $$\tau \left(\epsilon ,{\tilde{W}}_{n}\left(\frac{1}{2}\right)\right)=\Theta \left(n^{2}\log\epsilon^{-1}\right).$$

Finally, from (16), we obtain:

(21) $$T\left(\epsilon ,{\tilde{W}}_{n}\left(\frac{1}{2}\right)\right)∼\frac{2n^{3}\log\epsilon^{-1}}{\pi^{2}},$$

and hence, $T\left(\epsilon ,{\tilde{W}}_{n}\left(\frac{1}{2}\right)\right)=\Theta \left(n^{3}\log\epsilon^{-1}\right)$ .

### 2.2. Convergence Time of the Fastest Constant Edge Weights Algorithm

In [2], the authors consider the real symmetric weighting matrices $W_{n}\left(\rho \right)$ given by:

(22) $${\left[W_{n}\left(\rho \right)\right]}_{j,k}:=\left\{\begin{array}{cc} \rho & \mathrm{if}j\neq k,\mathrm{and}v_{j}\mathrm{and}v_{k}\mathrm{are}\mathrm{connected},\\ 1-d_{j}\rho & \mathrm{if}j=k,\\ 0 & \mathrm{otherwise}, \end{array}\right.$$

where $d_{j}$ denotes the degree of the sensor $v_{j}$ (i.e., the number of sensors different from $v_{j}$ connected to $v_{j}$ ).

Observe that the weighting matrices of the fastest LTI distributed averaging algorithms for symmetric weights given in Section 2.1 for a cycle and a path, namely ${\overset{\circ }{W}}_{n}\left(\gamma_{0}\right)$ and ${\tilde{W}}_{n}\left(\frac{1}{2}\right)$ , can be regarded as $W_{n}\left(\rho \right)$ in (22) taking $\rho =\gamma_{0}$ and $\rho =\frac{1}{2}$ , respectively. Therefore, the closed-form expression for the ϵ -convergence time of the fastest constant edge weights algorithm is given by Theorem 2 on a cycle and by Theorem 4 on a path. That is, the ϵ -convergence time of the fastest constant edge weights algorithm and the ϵ -convergence time of the fastest LTI distributed averaging algorithm for symmetric weights is the same on a cycle and on a path.

### 2.3. Convergence Time of the Maximum-Degree Weights Algorithm and of the Metropolis–Hastings Algorithm

For the maximum-degree weights algorithm [3], the weighting matrix considered is the real symmetric matrix $W_{n}\left(\rho \right)$ in (22) with:

(23) $$\rho =\frac{1}{1+\max_{j\in \{1,\ldots ,n\}}d_{j}}.$$

On the other hand, for the Metropolis–Hastings algorithm [3], the entries of the weighting matrix $W_{n}$ are given by:

(24) $${\left[W_{n}\right]}_{j,k}=\left\{\begin{array}{cc} \frac{{\left[A\right]}_{j,k}}{1+\max\{d_{j},d_{k}\}} & \mathrm{if}j\neq k,\\ 1-\sum_{h\in \{1,\ldots ,n\}\setminus \{j\}}{\left[W_{n}\right]}_{j,h} & \mathrm{if}j=k, \end{array}\right.$$

where *A* is the adjacency matrix of the network, that is *A* is the n×n real symmetric matrix given by:

(25) $${\left[A\right]}_{j,k}=\left\{\begin{array}{cc} 1 & \mathrm{if}j\neq k,\mathrm{and}v_{j}\mathrm{and}v_{k}\mathrm{are}\mathrm{connected},\\ 0 & \mathrm{otherwise}. \end{array}\right.$$

#### 2.3.1. The Cycle

Observe that the weighting matrices of the maximum-degree weights algorithm and the Metropolis–Hastings algorithm for a cycle with *n* sensors can be regarded as ${\overset{\circ }{W}}_{n}\left(\gamma \right)$ in (4) taking $\gamma =\frac{1}{3}$ .

We now give a closed-form expression for the ϵ -convergence time of the maximum-degree weights algorithm and of the Metropolis–Hastings algorithm on a cycle. We also study the asymptotic behavior of this convergence time as the number of sensors of the cycle grows.

**Theorem** **5.** *Consider ϵ∈(0,1) and n∈N , with n>3 . Then:*

(26) $$\tau \left(\epsilon ,{\overset{\circ }{W}}_{n}\left(\frac{1}{3}\right)\right)=\left\lceil \frac{\log\epsilon^{-1}}{-\log\frac{1+2\cos\frac{2\pi }{n}}{3}}\right\rceil .$$

*Moreover,*

(27) $$\tau \left(\epsilon ,{\overset{\circ }{W}}_{n}\left(\frac{1}{3}\right)\right)∼\frac{3n^{2}\log\epsilon^{-1}}{4\pi^{2}},$$

*and therefore,*

(28) $$\tau \left(\epsilon ,{\overset{\circ }{W}}_{n}\left(\frac{1}{3}\right)\right)=\Theta \left(n^{2}\log\epsilon^{-1}\right).$$

**Proof.**  Combining (A29) and (A30), we obtain:

(29) $$\begin{array}{c} {∥{\overset{\circ }{W}}_{n}\left(\frac{1}{3}\right)-P_{n}∥}_{2}=\frac{1+2\cos\frac{2\pi }{n}}{3}, \end{array}$$

and applying [2] (Theorem 1) and Theorem A1, (26) holds. The rest of the proof runs as the proof of Theorem 2.  ☐

Since the number of transmissions per iteration on a cycle with *n* sensors is *n* for both algorithms, the total number of transmissions required for $\tau \left(\epsilon ,{\overset{\circ }{W}}_{n}\left(\frac{1}{3}\right)\right)$ iterations is $T\left(\epsilon ,{\overset{\circ }{W}}_{n}\left(\frac{1}{3}\right)\right):=n\tau \left(\epsilon ,{\overset{\circ }{W}}_{n}\left(\frac{1}{3}\right)\right)$ . From Theorem 5, we obtain:

(30) $$T\left(\epsilon ,{\overset{\circ }{W}}_{n}\left(\frac{1}{3}\right)\right)∼\frac{3n^{3}\log\epsilon^{-1}}{4\pi^{2}},$$

and thus, $T\left(\epsilon ,{\overset{\circ }{W}}_{n}\left(\frac{1}{3}\right)\right)=\Theta \left(n^{3}\log\epsilon^{-1}\right)$ .

#### 2.3.2. The Path

Observe that the weighting matrices of the maximum-degree weights algorithm and of the Metropolis–Hastings algorithm for a path with *n* sensors can be regarded as ${\tilde{W}}_{n}\left(\alpha \right)$ in (11) taking $\alpha =\frac{1}{3}$ .

We now give a closed-form expression for the ϵ -convergence time of the maximum-degree weights algorithm and of the Metropolis–Hastings algorithm on a path. We also study the asymptotic behavior of this convergence time as the number of sensors of the path grows.

**Theorem** **6.** *Consider ϵ∈(0,1) and n∈N , with n>3 . Then:*

(31) $$\tau \left(\epsilon ,{\tilde{W}}_{n}\left(\frac{1}{3}\right)\right)=\left\lceil \frac{\log\epsilon^{-1}}{-\log\frac{1+2\cos\frac{\pi }{n}}{3}}\right\rceil .$$

*Moreover,*

(32) $$\tau \left(\epsilon ,{\tilde{W}}_{n}\left(\frac{1}{3}\right)\right)∼\frac{3n^{2}\log\epsilon^{-1}}{\pi^{2}},$$

*and therefore,*

(33) $$\tau \left(\epsilon ,{\tilde{W}}_{n}\left(\frac{1}{3}\right)\right)=\Theta \left(n^{2}\log\epsilon^{-1}\right).$$

**Proof.**  Combining (A63) and [4] (Lemma 1), we obtain:

(34) $$\begin{array}{c} {∥{\tilde{W}}_{n}\left(\frac{1}{3}\right)-P_{n}∥}_{2}=\frac{1}{3}+\frac{2}{3}\cos\frac{\pi }{n}, \end{array}$$

and applying [2] (Theorem 1) and Theorem A1, (31) holds. The rest of the proof runs as the proof of Theorem 2.  ☐

Since the number of transmissions per iteration on a path with *n* sensors is *n* for both algorithms, the total number of transmissions required for $\tau \left(\epsilon ,{\tilde{W}}_{n}\left(\frac{1}{3}\right)\right)$ iterations is $T\left(\epsilon ,{\tilde{W}}_{n}\left(\frac{1}{3}\right)\right):=n\tau \left(\epsilon ,{\tilde{W}}_{n}\left(\frac{1}{3}\right)\right)$ . From Theorem 6, we obtain:

(35) $$T\left(\epsilon ,{\tilde{W}}_{n}\left(\frac{1}{3}\right)\right)∼\frac{3n^{3}\log\epsilon^{-1}}{\pi^{2}},$$

and thus, $T\left(\epsilon ,{\tilde{W}}_{n}\left(\frac{1}{3}\right)\right)=\Theta \left(n^{3}\log\epsilon^{-1}\right)$ .

## 3. Convergence Time of Randomized Linear Distributed Averaging Algorithms

### 3.1. Lower and Upper Bounds for the Convergence Time of the Pairwise Gossip Algorithm

In the literature, we have found two different definitions for the convergence time of a randomized linear distributed averaging algorithm (see [6,7]). In this subsection, we consider the definition of ϵ -convergence time for a randomized linear distributed averaging algorithm given in [6]:

(36) $$\tau \left(\epsilon ,{\left\{W\left(t\right)\right\}}_{t\geq 0}\right):=\underset{x\left(0\right)\neq 0_{n\times 1}}{\sup}\inf\left\{t:\mathrm{Pr}\left(\frac{∥x\left(t\right)-P_{n}{x\left(0\right)∥}_{2}}{{∥x\left(0\right)∥}_{2}}\geq \epsilon \right)\leq \epsilon \right\},$$

where ϵ∈(0,1) and Pr denotes probability.

We prove in Theorem A1 (Appendix A) that the definitions of ϵ -convergence time in (3) and (36) coincide when applied to deterministic LTI distributed averaging algorithms with symmetric weights (in particular, the four algorithms considered in Section 2). For those algorithms, we also obtain from Theorem A1 that:

(37) $$\tau \left(\frac{1}{e},W\right)=\left\lceil \tau^{\prime }\left(W\right)\right\rceil ,$$

where $\tau^{\prime }\left(W\right)$ denotes the definition of convergence time given in [2].

We recall here that in the pairwise gossip algorithm [6], only two sensors interchange information at each time instant *t*. These two sensors $v_{j_{t}}$ and $v_{k_{t}}$ are randomly selected at each time instant *t*, and the weighting matrix W(t) , which we denote by $W_{P}\left(t\right)$ , is the symmetric matrix given by:

(38) $${\left[W_{P}\left(t\right)\right]}_{j,k}=\left\{\begin{array}{cc} \frac{1}{2} & \mathrm{if}j,k\in \{j_{t},k_{t}\},\\ 1 & \mathrm{if}j=k\notin \{j_{t},k_{t}\},\\ 0 & \mathrm{otherwise}, \end{array}\right.$$

for all j,k∈{1,…,n} .

In [6], a lower and an upper bound for the ϵ -convergence time of the pairwise gossip algorithm were introduced. We now give a closed-form expression for those bounds on a cycle and on a path, and we study their asymptotic behavior as the number of sensors of the network grows.

#### 3.1.1. The Cycle

**Theorem** **7.** *Consider ϵ∈(0,1) and n∈N , with n>3 . Suppose that ${\overset{\circ }{W}}_{P}\left(t\right)$ is the weighting matrix of the pairwise gossip algorithm given in (38) on a cycle with n sensors, where the edge $\left\{v_{j_{t}},v_{k_{t}}\right\}$ is randomly selected at each time instant t∈N∪{0} with probability $\frac{1}{n}$ . Then:*

(39) $$\frac{1}{2}{\overset{\circ }{l}}_{P}\left(\epsilon \right)\leq \tau \left(\epsilon ,{\left\{{\overset{\circ }{W}}_{P}\left(t\right)\right\}}_{t\geq 0}\right)\leq 3{\overset{\circ }{l}}_{P}\left(\epsilon \right),$$

*with:*

(40) $${\overset{\circ }{l}}_{P}\left(\epsilon \right)=\frac{\log\epsilon^{-1}}{-\log\left(1+\frac{1}{n}\left(\cos\frac{2\pi }{n}-1\right)\right)}.$$

*Moreover,*

(41) $${\overset{\circ }{l}}_{P}\left(\epsilon \right)∼\frac{n^{3}\log\epsilon^{-1}}{2\pi^{2}}$$

*and:*

(42) $$\tau \left(\epsilon ,{\left\{{\overset{\circ }{W}}_{P}\left(t\right)\right\}}_{t\geq 0}\right)=\Theta \left(n^{3}\log\epsilon^{-1}\right)=\tau \left(\epsilon ,{\overset{\circ }{W}}_{n}\left(\frac{1}{2n}\right)\right),$$

**Proof.**  The entries of the expectation of ${\overset{\circ }{W}}_{P}\left(0\right)$ are given by:

(43) $${\left[E\left({\overset{\circ }{W}}_{P}\left(0\right)\right)\right]}_{j,k}=\left\{\begin{array}{cc} \frac{1}{2n} & \mathrm{if}j-k\in \{-1,1\},\\ \frac{1}{2n} & \mathrm{if}j-k\in \{1-n,n-1\},\\ \frac{1}{n}\left(n-1\right) & \mathrm{if}j=k,\\ 0 & \mathrm{otherwise}, \end{array}\right.$$

for all j,k∈{1,…,n} . Thus, $E\left({\overset{\circ }{W}}_{P}\left(0\right)\right)={\overset{\circ }{W}}_{n}\left(\frac{1}{2n}\right)$ . Therefore, combining (A29) and [6] (Theorem 3), we obtain (39). The rest of the proof runs as the proof of Theorem 2.  ☐

Since the number of transmissions per iteration on a cycle with *n* sensors is two for the pairwise gossip algorithm, the total number of transmissions required for $\tau \left(\epsilon ,{\left\{{\overset{\circ }{W}}_{P}\left(t\right)\right\}}_{t\geq 0}\right)$ iterations is $T\left(\epsilon ,{\left\{{\overset{\circ }{W}}_{P}\left(t\right)\right\}}_{t\geq 0}\right):=2\tau \left(\epsilon ,{\left\{{\overset{\circ }{W}}_{P}\left(t\right)\right\}}_{t\geq 0}\right)$ . From Theorem 7, we obtain ${\overset{\circ }{l}}_{P}\left(\epsilon \right)\leq \;T\left(\epsilon ,{\left\{{\overset{\circ }{W}}_{P}\left(t\right)\right\}}_{t\geq 0}\right)\leq 6{\overset{\circ }{l}}_{P}\left(\epsilon \right)$ and $T\left(\epsilon ,{\left\{{\overset{\circ }{W}}_{P}\left(t\right)\right\}}_{t\geq 0}\right)=\Theta \left(n^{3}\log\epsilon^{-1}\right)$ .

#### 3.1.2. The Path

**Theorem** **8.** *Consider ϵ∈(0,1) and n∈N , with n>3 . Suppose that ${\tilde{W}}_{P}\left(t\right)$ is the weighting matrix of the pairwise gossip algorithm given in (38) on a path with n sensors, where the edge $\left\{v_{j_{t}},v_{k_{t}}\right\}$ is randomly selected at each time instant t∈N∪{0} with probability $\frac{1}{n-1}$ . Then:*

(44) $$\frac{1}{2}{\tilde{l}}_{P}\left(\epsilon \right)\leq \tau \left(\epsilon ,{\left\{{\tilde{W}}_{P}\left(t\right)\right\}}_{t\geq 0}\right)\leq 3{\tilde{l}}_{P}\left(\epsilon \right),$$

*with:*

(45) $${\tilde{l}}_{P}\left(\epsilon \right)=\frac{\log\epsilon^{-1}}{-\log\left(1+\frac{1}{n-1}\left(\cos\frac{\pi }{n}-1\right)\right)}.$$

*Moreover,*

(46) $${\tilde{l}}_{P}\left(\epsilon \right)∼\frac{2n^{3}\log\epsilon^{-1}}{\pi^{2}}$$

*and:*

(47) $$\tau \left(\epsilon ,{\left\{{\tilde{W}}_{P}\left(t\right)\right\}}_{t\geq 0}\right)=\Theta \left(n^{3}\log\epsilon^{-1}\right)=\tau \left(\epsilon ,{\tilde{W}}_{n}\left(\frac{1}{2n-2}\right)\right).$$

**Proof.**  The entries of the expectation of ${\tilde{W}}_{P}\left(0\right)$ are given by:

(48) $${\left[E\left({\tilde{W}}_{P}\left(0\right)\right)\right]}_{j,k}=\left\{\begin{array}{cc} \frac{1}{2n-2} & \mathrm{if}j-k\in \{-1,1\},\\ 1-\frac{1}{n-1} & \mathrm{if}j=k,j\neq 1\mathrm{and}j\neq n,\\ 1-\frac{1}{2n-2} & \mathrm{if}j=k,j\in \{1,n\},\\ 0 & \mathrm{otherwise}, \end{array}\right.$$

for all j,k∈{1,…,n} . Thus, $E\left({\tilde{W}}_{P}\left(0\right)\right)={\tilde{W}}_{n}\left(\frac{1}{2n-2}\right)$ . Therefore, combining (A63) and [6] (Theorem 3), we obtain (44). The rest of the proof runs as the proof of Theorem 2.  ☐

Since the number of transmissions per iteration on a path with *n* sensors is two for the pairwise gossip algorithm, the total number of transmissions required for $\tau \left(\epsilon ,{\left\{{\tilde{W}}_{P}\left(t\right)\right\}}_{t\geq 0}\right)$ iterations is $T\left(\epsilon ,{\left\{{\tilde{W}}_{P}\left(t\right)\right\}}_{t\geq 0}\right):=2\tau \left(\epsilon ,{\left\{{\tilde{W}}_{P}\left(t\right)\right\}}_{t\geq 0}\right)$ . From Theorem 8, we obtain ${\tilde{l}}_{P}\left(\epsilon \right)\leq T\left(\epsilon ,{\left\{{\tilde{W}}_{P}\left(t\right)\right\}}_{t\geq 0}\right)\leq 6{\tilde{l}}_{P}\left(\epsilon \right)$ and $T\left(\epsilon ,{\left\{{\tilde{W}}_{P}\left(t\right)\right\}}_{t\geq 0}\right)=\Theta \left(n^{3}\log\epsilon^{-1}\right)$ .

### 3.2. Lower and Upper Bounds for the Convergence Time of the Broadcast Gossip Algorithm

We begin this subsection with the definition of ϵ -convergence time for a randomized linear distributed averaging algorithm given in [7] (Equation (42)):

(49) $$\tau \left(\epsilon ,{\left\{W\left(t\right)\right\}}_{t\geq 0}\right):=\underset{x\left(0\right)\neq P_{n}x\left(0\right)}{\sup}\inf\left\{t:\mathrm{Pr}\left(\frac{∥x\left(t\right)-P_{n}{x\left(t\right)∥}_{2}}{∥x\left(0\right)-P_{n}{x\left(0\right)∥}_{2}}\geq \epsilon \right)\leq \epsilon \right\},$$

where ϵ∈(0,1) .

It can be proven that the definitions of ϵ -convergence time in (36) and (49) coincide when applied to algorithms in which the matrix W(t) satisfies $W\left(t\right)P_{n}=P_{n}W\left(t\right)=P_{n}$ for all t∈N∪{0} (in particular, the pairwise gossip algorithm and deterministic LTI distributed averaging algorithms with symmetric weights).

Observe that (49) is actually a definition for the convergence time of linear distributed consensus algorithms, not only of linear distributed averaging algorithms.

We recall here that in the broadcast gossip algorithm, a single sensor broadcasts at each time instant *t*. This sensor $v_{j_{t}}$ is randomly selected at each time instant *t* with probability $\frac{1}{n}$ , and the weighting matrix W(t) is given by:

(50) $${\left[W\left(t\right)\right]}_{j,k}=\left\{\begin{array}{cc} 1 & \mathrm{if}j=k\mathrm{and}{\left[A\right]}_{j,j_{t}}=0,\\ \varphi & \mathrm{if}j=k\mathrm{and}{\left[A\right]}_{j,j_{t}}=1,\\ 1-\varphi & \mathrm{if}k=j_{t}\mathrm{and}{\left[A\right]}_{j,j_{t}}=1,\\ 0 & \mathrm{otherwise}, \end{array}\right.$$

for all j,k∈{1,…,n} , where φ∈(0,1) and *A* is the adjacency matrix of the network. We denote by $W_{B}\left(t\right)$ the weighting matrix in (50) when φ is the optimal parameter: $\varphi_{0}$ (see [7] (Section V)).

In [7], a lower and an upper bound for the ϵ -convergence time of the broadcast gossip algorithm were introduced. We now give a closed-form expression for $\varphi_{0}$ and for those bounds on a cycle and on a path. We also study the asymptotic behavior of the bounds as the number of sensors of the network grows.

#### 3.2.1. The Cycle

**Theorem** **9.** *Consider ϵ∈(0,1) and n∈N , with n>3 . Suppose that ${\overset{\circ }{W}}_{B}\left(t\right)$ is the weighting matrix in (50) when the network is a cycle with n sensors and φ is the optimal parameter: ${\overset{\circ }{\varphi }}_{0}$ . Then:*

(51) $${\overset{\circ }{\varphi }}_{0}=1-\frac{n}{2\left(n+\cos\frac{2\pi }{n}-1\right)}$$

*and:*

(52) $${\overset{\circ }{l}}_{B}\left(\epsilon \right)\leq \tau \left(\epsilon ,{\left\{{\overset{\circ }{W}}_{B}\left(t\right)\right\}}_{t\geq 0}\right)\leq 6{\overset{\circ }{l}}_{B}\left(\epsilon \right),$$

*with:*

(53) $${\overset{\circ }{l}}_{B}\left(\epsilon \right)=\frac{\log\epsilon^{-1}}{-2\log\left(\frac{n+2\cos\frac{2\pi }{n}-2}{n+\cos\frac{2\pi }{n}-1}\right)}.$$

*Moreover,*

(54) $${\overset{\circ }{l}}_{B}\left(\epsilon \right)∼\frac{n^{3}\log\epsilon^{-1}}{4\pi^{2}},$$

*and:*

(55) $$\tau \left(\epsilon ,{\left\{{\overset{\circ }{W}}_{B}\left(t\right)\right\}}_{t\geq 0}\right)=\Theta \left(n^{3}\log\epsilon^{-1}\right)=\tau \left(\epsilon ,{\overset{\circ }{W}}_{n}\left(\frac{1-{\overset{\circ }{\varphi }}_{0}}{n}\right)\right).$$

**Proof.**  See Appendix E.  ☐

Since the number of transmissions per iteration on a cycle with *n* sensors is one for the broadcast gossip algorithm, the total number of transmissions required for $\tau \left(\epsilon ,{\left\{{\overset{\circ }{W}}_{B}\left(t\right)\right\}}_{t\geq 0}\right)$ iterations is $T\left(\epsilon ,{\left\{{\overset{\circ }{W}}_{B}\left(t\right)\right\}}_{t\geq 0}\right):=\tau \left(\epsilon ,{\left\{{\overset{\circ }{W}}_{B}\left(t\right)\right\}}_{t\geq 0}\right)$ . From Theorem 9, we obtain ${\overset{\circ }{l}}_{B}\left(\epsilon \right)\leq T\left(\epsilon ,{\left\{{\overset{\circ }{W}}_{B}\left(t\right)\right\}}_{t\geq 0}\right)\leq 6{\overset{\circ }{l}}_{B}\left(\epsilon \right)$ and $T\left(\epsilon ,{\left\{{\overset{\circ }{W}}_{B}\left(t\right)\right\}}_{t\geq 0}\right)=\Theta \left(n^{3}\log\epsilon^{-1}\right)$ .

#### 3.2.2. The Path

**Theorem** **10.** *Consider ϵ∈(0,1) and n∈N , with n>3 . Suppose that ${\tilde{W}}_{B}\left(t\right)$ is the weighting matrix in (50) when the network is a path with n sensors and φ is the optimal parameter: ${\tilde{\varphi }}_{0}$ . Then:*

(56) $${\tilde{\varphi }}_{0}=1-\frac{n}{2\left(n+\cos\frac{\pi }{n}-1\right)}$$

*and:*

(57) $${\tilde{l}}_{B}\left(\epsilon \right)\leq \tau \left(\epsilon ,{\left\{{\tilde{W}}_{B}\left(t\right)\right\}}_{t\geq 0}\right)\leq 6{\tilde{l}}_{B}\left(\epsilon \right),$$

*with:*

(58) $${\tilde{l}}_{B}\left(\epsilon \right)=\frac{\log\epsilon^{-1}}{-2\log\left(\frac{n+2\cos\frac{\pi }{n}-2}{n+\cos\frac{\pi }{n}-1}\right)}.$$

*Moreover,*

(59) $${\tilde{l}}_{B}\left(\epsilon \right)∼\frac{n^{3}\log\epsilon^{-1}}{\pi^{2}},$$

*and:*

(60) $$\tau \left(\epsilon ,{\left\{{\tilde{W}}_{B}\left(t\right)\right\}}_{t\geq 0}\right)=\Theta \left(n^{3}\log\epsilon^{-1}\right)=\tau \left(\epsilon ,{\tilde{W}}_{n}\left(\frac{1-{\overset{\circ }{\varphi }}_{0}}{n}\right)\right).$$

**Proof.**  See Appendix F.  ☐

Since the number of transmissions per iteration on a path with *n* sensors is one for the broadcast gossip algorithm, the total number of transmissions required for $\tau \left(\epsilon ,{\left\{{\tilde{W}}_{B}\left(t\right)\right\}}_{t\geq 0}\right)$ iterations is $T\left(\epsilon ,{\left\{{\tilde{W}}_{B}\left(t\right)\right\}}_{t\geq 0}\right):=\tau \left(\epsilon ,{\left\{{\tilde{W}}_{B}\left(t\right)\right\}}_{t\geq 0}\right)$ . From Theorem 10, we obtain ${\tilde{l}}_{B}\left(\epsilon \right)\leq T\left(\epsilon ,{\left\{{\tilde{W}}_{B}\left(t\right)\right\}}_{t\geq 0}\right)\leq 6{\tilde{l}}_{B}\left(\epsilon \right)$ and $T\left(\epsilon ,{\left\{{\tilde{W}}_{B}\left(t\right)\right\}}_{t\geq 0}\right)=\Theta \left(n^{3}\log\epsilon^{-1}\right)$ .

## 4. Discussion

As in this paper we have used the same definition of converge time for both deterministic and randomized linear distributed averaging algorithms (namely, the one in (49)), the results given in Section 2 and Section 3 allow us to compare the considered algorithms on a cycle and on a path in terms of convergence time and, consequently, in terms of the number of transmissions required, as well. In particular, these results show the following:

- The behavior of the considered deterministic linear distributed averaging algorithms is as good as the behavior of the considered randomized ones in terms of the number of transmissions required on a cycle and on a path with *n* sensors: $\Theta (n^{3}\log\epsilon^{-1})$ .
- For a large enough number of sensors and regardless of the considered distributed averaging algorithm, the number of transmissions required on a path is four times larger than the number of transmissions required on a cycle.

Furthermore, regarding the cycle, from (10), (30), (41) and (54), we obtain the following enlightening asymptotic equalities:

(61) $${\overset{\circ }{l}}_{B}\left(\epsilon \right)∼\frac{T(\epsilon ,{\overset{\circ }{W}}_{n}\left(\gamma_{0}\right))}{2}∼\frac{{\overset{\circ }{l}}_{P}\left(\epsilon \right)}{2}∼\frac{T\left(\epsilon ,{\overset{\circ }{W}}_{n}\left(\frac{1}{3}\right)\right)}{3},$$

and regarding the path, from (21), (35), (46) and (59), we obtain:

(62) $${\tilde{l}}_{B}\left(\epsilon \right)∼\frac{T\left(\epsilon ,{\tilde{W}}_{n}\left(\frac{1}{2}\right)\right)}{2}∼\frac{{\tilde{l}}_{P}\left(\epsilon \right)}{2}∼\frac{T\left(\epsilon ,{\tilde{W}}_{n}\left(\frac{1}{3}\right)\right)}{3}.$$

## 5. Numerical Examples

**Figure 2:**
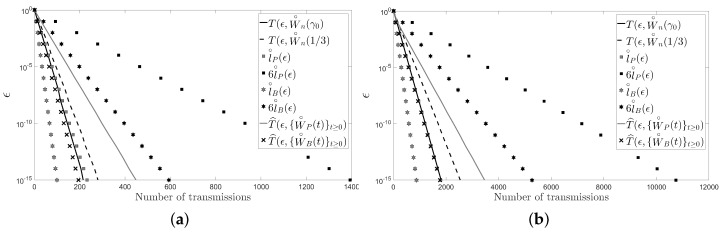
(**a**) A cycle with five sensors; (**b**) a cycle with 10 sensors.

**Figure 3:**
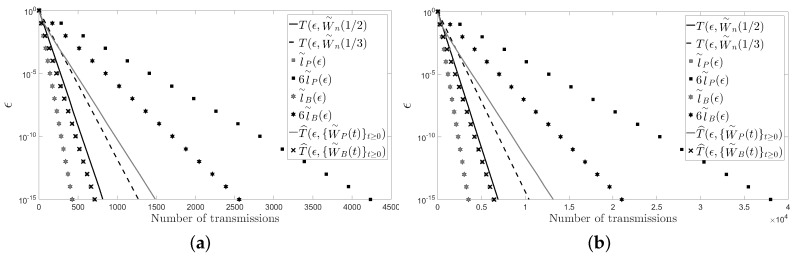
(**a**) A path with five sensors; (**b**) a path with 10 sensors.

For the numerical examples, we first consider a cycle and a path with five and 10 sensors. For each network topology, we present a figure: Figure 2 for the cycle and Figure 3 for the path. Figure 2 (resp. Figure 3) shows the number of transmissions of the fastest LTI distributed averaging algorithm for symmetric weights $T(\epsilon ,{\overset{\circ }{W}}_{n}\left(\gamma_{0}\right))$ (resp. $T(\epsilon ,{\tilde{W}}_{n}\left(1/2\right))$ ) and of the Metropolis–Hastings algorithm $T(\epsilon ,{\overset{\circ }{W}}_{n}\left(1/3\right))$ (resp. $T(\epsilon ,{\tilde{W}}_{n}\left(1/3\right))$ ) with $\epsilon \in (10^{-15},1)$ . The figure also shows the lower bound, ${\overset{\circ }{l}}_{P}\left(\epsilon \right)$ , and upper bound, $6{\overset{\circ }{l}}_{P}\left(\epsilon \right)$ , given for the number of transmissions of the pairwise gossip algorithm, and the lower bound, ${\overset{\circ }{l}}_{B}\left(\epsilon \right)$ , and upper bound, $6{\overset{\circ }{l}}_{B}\left(\epsilon \right)$ , given for the number of transmissions of the broadcast gossip algorithm (resp. ${\tilde{l}}_{P}\left(\epsilon \right)$ , $6{\tilde{l}}_{P}\left(\epsilon \right)$ , ${\tilde{l}}_{B}\left(\epsilon \right)$ and $6{\tilde{l}}_{B}\left(\epsilon \right)$ ). Furthermore, the figure shows the average number of transmissions of the pairwise gossip algorithm, $\hat{T}\left(\epsilon ,{\left\{{\overset{\circ }{W}}_{P}\left(t\right)\right\}}_{t\geq 0}\right)$ , and of the broadcast gossip algorithm, $\hat{T}\left(\epsilon ,{\left\{{\overset{\circ }{W}}_{B}\left(t\right)\right\}}_{t\geq 0}\right)$ , (resp. $\hat{T}\left(\epsilon ,{\left\{{\tilde{W}}_{P}\left(t\right)\right\}}_{t\geq 0}\right)$ and $\hat{T}\left(\epsilon ,{\left\{{\tilde{W}}_{B}\left(t\right)\right\}}_{t\geq 0}\right)$ ), that we have computed by using Monte Carlo simulations. In those simulations, we have performed 1000 repetitions of the corresponding algorithm for each $\epsilon \in (10^{-15},1)$ , and we have considered that the values measured by the sensors, $x_{j}\left(0\right)$ with j∈{1,…,n} , are independent identically distributed random variables with unit-variance, zero-mean and uniform distribution.

**Figure 4:**
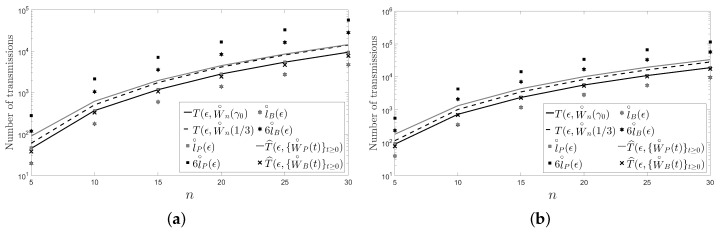
A cycle: (**a**) $\epsilon =10^{-3}$ ; (**b**) $\epsilon =10^{-6}$ .

**Figure 5:**
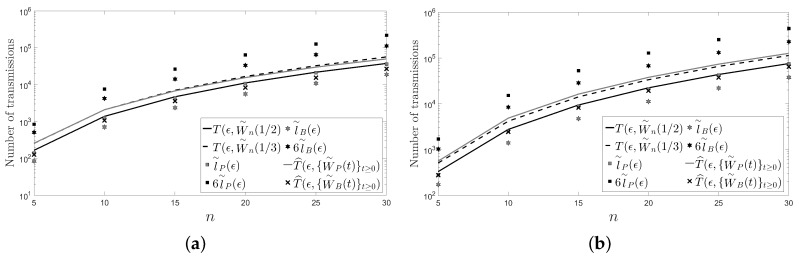
A path: (**a**) $\epsilon =10^{-3}$ ; (**a**) $\epsilon =10^{-6}$ .

In this section, we present another two figures: Figure 4 and Figure 5. Unlike in Figure 2 and Figure 3, in Figure 4 and Figure 5, we have fixed ϵ instead of the number of sensors *n* of the network. Specifically, we have chosen $\epsilon =10^{-3}$ and $\epsilon =10^{-6}$ with n∈{5,…,30} .

In the figures, it can be observed that the Metropolis–Hastings algorithm behaves on average better than the pairwise gossip algorithm in terms of the number of transmissions required on the considered networks. It can also be observed that the broadcast gossip algorithm behaves on average approximately equal to the fastest LTI distributed averaging algorithm for symmetric weights in terms of the number of transmissions required on those networks. However, we recall here that the broadcast gossip algorithm converges to a random consensus value instead of to the average consensus value, and it should be executed several times in order to get that average value in every sensor.

The figures also bear evidence of the asymptotic equalities given in (61) and in (62).

## 6. Conclusions

In this paper, we have studied the convergence time of six known linear distributed averaging algorithms. We have considered both deterministic (the fastest LTI distributed averaging algorithm for symmetric weights, the fastest constant edge weights algorithm, the maximum-degree weights algorithm and the Metropolis–Hastings algorithm) and randomized (the pairwise gossip algorithm and the broadcast gossip algorithm) linear distributed averaging algorithms. In the literature, we have not found closed-form expressions for the convergence time of the considered algorithms. We have computed closed-form expressions for the convergence time of the deterministic algorithms and closed-form upper bounds for the convergence time of the randomized algorithms on two common network topologies: the cycle and the path. Moreover, we have also computed a closed-form expression for the convergence time of the fastest LTI algorithm on a grid. From the computed closed-form formulas, we have studied the asymptotic behavior of the convergence time of the considered algorithms as the number of sensors of the considered networks grows.

Although there exist different definitions of convergence time in the literature, in this paper, we have proven that one of them (namely, the one in (49)) encompasses all the others for the algorithms here considered. As we have used the definition of converge time in (49) for both deterministic and randomized linear distributed averaging algorithms, the obtained closed-form formulas and asymptotic results allow us to compare the considered algorithms on cycles and paths in terms of convergence time and, consequently, in terms of the number of transmissions required, as well.

We now summarize the most remarkable conclusions:

- The best algorithm among the considered deterministic distributed averaging algorithms is not worse than the best algorithm among the considered randomized distributed averaging algorithms for cycles and paths.
- The weighting matrix of the fastest LTI distributed averaging algorithm for symmetric weights and the weighting matrix of the fastest constant edge weights algorithm are the same on cycles and on paths.
- The number of transmissions required on a path with *n* sensors is asymptotically four-times larger than the number of transmissions required on a cycle with the same number of sensors.
- The number of transmissions required grows as $n^{3}$ on cycles and on paths for the six algorithms considered.
- For the fastest LTI algorithm, the number of transmissions required grows as $n^{2}$ on a square grid of *n* sensors (i.e., $r=c=\sqrt{n}$ ).

A future research direction of this work would be to generalize the analysis presented in the paper to other network topologies. In particular, networks that can be decomposed into cycles and paths could be studied.

## Appendix A. Comparison of Several Definitions of Convergence Time

We begin by giving a property of the spectral norm. Its proof is implicit in the Appendix of [1].

**Lemma** **A1.** *Let B and P be two n×n real symmetric matrices with BP=P (or equivalently, PB=P ). Suppose that P is idempotent. Then:*

1. *
  $B^{t}P=P$ for all t∈N .*
2. *
  $B^{t}-P={\left(B-P\right)}^{t}$ for all t∈N .*
3. *
  $∥B^{t}{-P∥}_{2}={∥B-P∥}_{2}^{t}$ for all t∈N .*

We recall that an n×n matrix *A* is idempotent if and only if $A^{2}=A$ . An example of idempotent matrix is $P_{n}$ with n∈N , since ${\left[P_{n}^{2}\right]}_{j,k}=\sum_{h=1}^{n}{\left[P_{n}\right]}_{j,h}{\left[P_{n}\right]}_{h,k}=\sum_{h=1}^{n}\frac{1}{n}\frac{1}{n}=\frac{1}{n}={\left[P_{n}\right]}_{j,k}$ for all j,k∈{1,…n} .

The following result gives an eigenvalue decomposition for the matrix $P_{n}$ for all n∈N .

**Lemma** **A2.**
*If n∈N , then $P_{n}=V_{n}\mathrm{diag}\left(1,0,\ldots ,0\right)V_{n}^{*}$ , where $V_{n}$ is the n×n Fourier unitary matrix.*

**Proof.** From [13] (Lemma 2) or [14] (Lemma 3), we obtain that $V_{n}\mathrm{diag}\left(1,0,\ldots ,0\right)V_{n}^{*}$ is a circulant matrix with:

(A1)
$${\left[V_{n}\mathrm{diag}\left(1,0,\ldots ,0\right)V_{n}^{*}\right]}_{j,1}={\left[\frac{1}{\sqrt{n}}V_{n}\left(\begin{array}{c} 1\\ 0\\ \vdots \\ 0 \end{array}\right)\right]}_{j,1}=\frac{1}{\sqrt{n}}\sum_{k=1}^{n}{\left[V_{n}\right]}_{j,k}{\left[\left(\begin{array}{c} 1\\ 0\\ \vdots \\ 0 \end{array}\right)\right]}_{k,1}=\frac{1}{\sqrt{n}}{\left[V_{n}\right]}_{j,1}=\frac{1}{n}$$

for all j∈{1,…n} . Therefore, $V_{n}\mathrm{diag}\left(1,0,\ldots ,0\right)V_{n}^{*}=P_{n}$ .  ☐

We finish this subsection with a result regarding the ϵ -convergence time.

**Theorem** **A1.** *Let B be an n×n real symmetric matrix with $B\neq P_{n}$ and $B^{t}\to P_{n}$ . If ϵ∈(0,1) , then:*

(A2)mint0∈N:∥Btx−Pnx∥2∥x−Pnx∥2≤ϵ,∀t≥t0,∀x≠Pnx=maxx≠0n×1mint∈N:∥Btx−Pnx∥2∥x∥2≤ϵ(A3)=logϵ−1−logB−Pn2.

**Proof.** Let t∈N . We first prove that the following statements are equivalent:

1. $\frac{∥B^{t}x-P_{n}{x∥}_{2}}{∥x-P_{n}{x∥}_{2}}\leq \epsilon$ for all $x\neq P_{n}x$ .
2. $\frac{∥B^{t}x-P_{n}{x∥}_{2}}{{∥x∥}_{2}}\leq \epsilon$ for all $x\neq 0_{n\times 1}$ .

1⇒2 Fix $x\neq 0_{n\times 1}$ . If $x\neq P_{n}x$ , applying Lemma A2 yields:

(A4)
$$\begin{array}{cc} \frac{∥B^{t}x-P_{n}{x∥}_{2}}{{∥x∥}_{2}} & \;\leq \;\frac{\epsilon ∥x-P_{n}{x∥}_{2}}{{∥x∥}_{2}}\;=\;\frac{\epsilon ∥\left(I_{n}-P_{n}\right){x∥}_{2}}{{∥x∥}_{2}}\;\leq \;\epsilon {∥I_{n}-P_{n}∥}_{2}\;=\;\epsilon {∥V_{n}V_{n}^{*}-V_{n}\mathrm{diag}\left(1,0,\ldots ,0\right)V_{n}^{*}∥}_{2} \end{array}$$

(A5)
$$\begin{array}{c} =\epsilon {∥V_{n}I_{n}V_{n}^{*}-V_{n}\mathrm{diag}\left(1,0,\ldots ,0\right)V_{n}^{*}∥}_{2}=\epsilon {∥V_{n}\mathrm{diag}\left(0,1,\ldots ,1\right)V_{n}^{*}∥}_{2}=\epsilon , \end{array}$$

where $I_{n}$ is the n×n identity matrix. If $x=P_{n}x$ , from Lemma A2 and [2] (Theorem 1), we obtain:

(A6)
$$\frac{∥B^{t}x-P_{n}{x∥}_{2}}{{∥x∥}_{2}}=\frac{∥B^{t}P_{n}x-P_{n}{x∥}_{2}}{{∥x∥}_{2}}=\frac{∥P_{n}x-P_{n}{x∥}_{2}}{{∥x∥}_{2}}=0<\epsilon .$$

2⇒1 If $x\neq P_{n}x$ , then:

(A7)
$$\begin{array}{cc} \frac{∥B^{t}x-P_{n}{x∥}_{2}}{∥x-P_{n}{x∥}_{2}} & =\frac{∥B^{t}x-P_{n}x-P_{n}x+P_{n}{x∥}_{2}}{∥x-P_{n}{x∥}_{2}}=\frac{∥B^{t}x-B^{t}P_{n}x-P_{n}x+P_{n}^{2}{x∥}_{2}}{∥x-P_{n}{x∥}_{2}} \end{array}$$

(A8)
$$\begin{array}{c} =\frac{∥B^{t}\left(x-P_{n}x\right)-P_{n}\left(x-P_{n}x\right){∥}_{2}}{∥x-P_{n}{x∥}_{2}}\leq \epsilon . \end{array}$$

Consequently,

(A9)
$$\begin{array}{c} \min\left\{t_{0}\in N:\frac{∥B^{t}x-P_{n}{x∥}_{2}}{∥x-P_{n}{x∥}_{2}}\leq \epsilon ,\forall t\geq t_{0},\forall x\neq P_{n}x\right\} \end{array}$$

(A10)
$$\begin{array}{c} =\min\left\{t_{0}\in N:\frac{∥B^{t}x-P_{n}{x∥}_{2}}{{∥x∥}_{2}}\leq \epsilon ,\forall t\geq t_{0},\forall x\neq 0_{n\times 1}\right\} \end{array}$$

(A11)
$$\begin{array}{c} =\min\left\{t_{0}\in N:\max_{x\neq 0_{n\times 1}}\frac{∥B^{t}x-P_{n}{x∥}_{2}}{{∥x∥}_{2}}\leq \epsilon ,\forall t\geq t_{0}\right\} \end{array}$$

(A12)
$$\begin{array}{c} =\min\left\{t_{0}\in N:{∥B^{t}-P_{n}∥}_{2}\leq \epsilon ,\forall t\geq t_{0}\right\} \end{array}$$

(A13)
$$\begin{array}{c} =\min\left\{t_{0}\in N:{∥B-P_{n}∥}_{2}^{t}\leq \epsilon ,\forall t\geq t_{0}\right\} \end{array}$$

(A14)
$$\begin{array}{c} =\min\left\{t_{0}\in N:{∥B-P_{n}∥}_{2}^{t_{0}}\leq \epsilon \right\} \end{array}$$

(A15)
$$\begin{array}{c} =\min\left\{t_{0}\in N:\log(∥B-P_{n}{∥}_{2}^{t_{0}})\leq \log\epsilon \right\} \end{array}$$

(A16)
$$\begin{array}{c} =\min\left\{t_{0}\in N:t_{0}\log{∥B-P_{n}∥}_{2}\leq \log\epsilon \right\}=\min\left\{t_{0}\in N:t_{0}\geq \frac{\log\epsilon }{\log∥B-P_{n}{∥}_{2}}\right\} \end{array}$$

(A17)
$$\begin{array}{c} =\min\left\{t_{0}\in N:t_{0}\geq \frac{\log\epsilon^{-1}}{-\log∥B-P_{n}{∥}_{2}}\right\}=\left\lceil \frac{\log\epsilon^{-1}}{-\log{∥B-P_{n}∥}_{2}}\right\rceil . \end{array}$$

To prove (A10). we have used the equivalence 1⇔2 . To show (A12) and (A13), we have applied the definition of the spectral norm (see, e.g., [15] (pp. 603, 609)) and Assertion 3 of Lemma A1, respectively. To prove (A14), we have used [2] (Theorem 1) ( $∥B-P_{n}{∥}_{2}<1$ ). As:

(A18)mint0∈N:∥Btx−Pnx∥2∥x−Pnx∥2≤ϵ,∀t≥t0,∀x≠Pnx=mint∈N:∥B−Pn∥2t≤ϵ(A19)=mint∈N:∥Bt−Pn∥2≤ϵ,

we only need to show that $T_{1}=T_{2}$ to finish the proof, where:

(A20)
$$T_{1}=\min\left\{t\in N:\max_{x\neq 0_{n\times 1}}\frac{∥B^{t}x-P_{n}{x∥}_{2}}{{∥x∥}_{2}}\leq \epsilon \right\}$$

and:

(A21)
$$T_{2}=\max_{x\neq 0_{n\times 1}}t_{x},$$

with:

(A22)
$$t_{x}=\min\left\{t\in N:\frac{∥B^{t}x-P_{n}{x∥}_{2}}{{∥x∥}_{2}}\leq \epsilon \right\}.$$

Since:

(A23)
$$\frac{∥B^{T_{1}}x-P_{n}{x∥}_{2}}{{∥x∥}_{2}}\leq \epsilon \;\forall x\neq 0_{n\times 1}$$

we have $t_{x}\leq T_{1}$ for all $x\neq 0_{n\times 1}$ and, consequently, $T_{2}\leq T_{1}$ . If $\left\{\frac{∥B^{t}x-P_{n}{x∥}_{2}}{{∥x∥}_{2}}\right\}$ is a decreasing sequence for all $x\neq 0_{n\times 1}$ , then:

(A24)
$$\frac{∥B^{T_{2}}x-P_{n}{x∥}_{2}}{{∥x∥}_{2}}\leq \frac{∥B^{t_{x}}x-P_{n}{x∥}_{2}}{{∥x∥}_{2}}\leq \epsilon \;\forall x\neq 0_{n\times 1}$$

and therefore,

(A25)
$$\max_{x\neq 0_{n\times 1}}\frac{∥B^{T_{2}}x-P_{n}{x∥}_{2}}{{∥x∥}_{2}}\leq \epsilon$$

and $T_{1}\leq T_{2}$ . Thus, if we prove that these sequences are decreasing, the proof is complete. Given $x\neq 0_{n\times 1}$ , from Lemma A1 and [2] (Theorem 1), we conclude that:

(A26)
$$\frac{∥B^{t+1}x-P_{n}{x∥}_{2}}{{∥x∥}_{2}}\;=\;\frac{∥{\left(B-P_{n}\right)}^{t+1}{x∥}_{2}}{{∥x∥}_{2}}\;\leq \;\frac{∥\left(B-P_{n}\right){∥}_{2}{∥{\left(B-P_{n}\right)}^{t}x∥}_{2}}{{∥x∥}_{2}}\;\leq \;\frac{∥{\left(B-P_{n}\right)}^{t}{x∥}_{2}}{{∥x∥}_{2}}\;=\;\frac{∥B^{t}x-P_{n}{x∥}_{2}}{{∥x∥}_{2}}$$

for all t∈N . To prove the two equalities in (A26), we have used Assertion 2 of Lemma A1. To show the first inequality in (A26), we have applied a well-known inequality on the spectral norm (see, e.g., [15] (p. 611)), and to prove the second inequality in (A26), we have used [2] (Theorem 1) ( $∥B-P_{n}{∥}_{2}<1$ ).  ☐

## Appendix B. Proof of Theorem 1

Let $B(\gamma_{1},\ldots ,\gamma_{n})$ be the n×n real symmetric matrix given by:

(A27)
$$\left(\begin{array}{ccccccc} 1\;\;-\;\;\gamma_{1}\;\;-\;\;\gamma_{n} & \gamma_{1} & 0 & \cdots & 0 & 0 & \gamma_{n}\\ \gamma_{1} & 1\;\;-\;\;\gamma_{1}\;\;-\;\;\gamma_{2} & \gamma_{2} & \cdots & 0 & 0 & 0\\ 0 & \gamma_{2} & 1\;\;-\;\;\gamma_{2}\;\;-\;\;\gamma_{3} & \cdots & 0 & 0 & 0\\ \vdots & \ddots & \ddots & \ddots & \ddots & \vdots & \vdots \\ 0 & 0 & 0 & \cdots & 1\;\;-\;\;\gamma_{n-3}\;\;-\;\;\gamma_{n-2} & \gamma_{n-2} & 0\\ 0 & 0 & 0 & \cdots & \gamma_{n-2} & 1\;\;-\;\;\gamma_{n-2}\;\;-\;\;\gamma_{n-1} & \gamma_{n-1}\\ \gamma_{n} & 0 & 0 & \cdots & 0 & \gamma_{n-1} & 1\;\;-\;\;\gamma_{n-1}\;\;-\;\;\gamma_{n} \end{array}\right).$$

Observe that the matrix in (A27) satisfies $B\left(\gamma_{1},\ldots ,\gamma_{n}\right)P_{n}=P_{n}$ .

We define the function $f:\;R^{n}\mapsto \left[0,\infty \right)$ as $f\left(\gamma_{1},\ldots ,\gamma_{n}\right):={∥B\left(\gamma_{1},\ldots ,\gamma_{n}\right)-P_{n}∥}_{2}$ . We next prove that:

(A28)
$${∥{\overset{\circ }{W}}_{n}\left(\gamma_{0}\right)-P_{n}∥}_{2}\leq {∥B\left(\gamma_{1},\ldots ,\gamma_{n}\right)-P_{n}∥}_{2}\;\forall \gamma_{1},\ldots ,\gamma_{n}\in R$$

Observe that ${\overset{\circ }{W}}_{n}\left(\gamma_{0}\right)=B\left(\gamma_{0},\ldots ,\gamma_{0}\right)$ . As ${\overset{\circ }{W}}_{n}\left(\gamma \right)$ is circulant, its eigenvalues are (see, e.g., [16] (Equation (3.7)) or [17] (Equation (5.2))):

(A29)
$$a_{j}:=1+2\gamma \left(\cos\frac{2\pi (j-1)}{n}-1\right),\;j\in \left\{1,\ldots ,n\right\}$$

Let $V_{n}=\left(v_{1}\left|\cdots \right|v_{n}\right)$ be the n×n Fourier unitary matrix. It is well known (see, e.g., [16] (Equation (3.11)) or [17] (Lemma 5.1)) that $v_{j}$ is an unit eigenvector of $\overset{\circ }{W}\left(\gamma \right)$ associated with the eigenvalue $a_{j}$ for all j∈{1,…,n} . From Lemma A2:

(A30)
$${∥{\overset{\circ }{W}}_{n}\left(\gamma \right)-P_{n}∥}_{2}=\max\{|a_{j}\left|:j\in \left\{2,\ldots ,n\right\}\right\}.$$

Case 1: Assume that *n* is even. Then,

(A31)
$${∥\overset{\circ }{W}\left(\gamma_{0}\right)-P_{n}∥}_{2}=a_{2}=-a_{\frac{n}{2}+1}=a_{n}=\frac{1+\cos\frac{2\pi }{n}}{3-\cos\frac{2\pi }{n}}\in \left(0,1\right).$$

Therefore, $y_{2}=\frac{\sqrt{2}}{2}\left(v_{n}+v_{2}\right)$ and $y_{n}=\frac{\sqrt{2}}{2\sqrt{-1}}\left(v_{n}-v_{2}\right)$ are unit eigenvectors of $\overset{\circ }{W}\left(\gamma_{0}\right)$ associated to $a_{2}=a_{n}$ . As:

(A32)
$$\begin{array}{cc} {\left[y_{2}\right]}_{j,1} & =\sqrt{\frac{2}{n}}\cos\frac{2\pi (j-1)}{n}, \end{array}$$

(A33)
$$\begin{array}{cc} {\left[y_{n}\right]}_{j,1} & =\sqrt{\frac{2}{n}}\sin\frac{2\pi (j-1)}{n}, \end{array}$$

(A34)
$$\begin{array}{cc} {\left[v_{\frac{n}{2}+1}\right]}_{j,1} & =\sqrt{\frac{1}{n}}{\left(-1\right)}^{j-1} \end{array}$$

for all j∈{1,…,n} , from [4] (Theorem 1), we obtain three subgradients of *f* at $\left(\gamma_{0},\ldots ,\gamma_{0}\right)\in R^{n}$ , namely $g_{1}$ , $g_{2}$ and $g_{3}$ , given by:

(A35)
$$\begin{array}{cc} {\left[g_{1}\right]}_{j,1} & =-\frac{2}{n}{\left(\cos\frac{2\pi (j-1)}{n}-\cos\frac{2\pi j}{n}\right)}^{2}, \end{array}$$

(A36)
$$\begin{array}{cc} {\left[g_{3}\right]}_{j,1} & =-\frac{2}{n}{\left(\sin\frac{2\pi (j-1)}{n}-\sin\frac{2\pi j}{n}\right)}^{2}, \end{array}$$

(A37)
$$\begin{array}{cc} {\left[g_{2}\right]}_{j,1} & =\frac{4}{n} \end{array}$$

for all j∈{1,…,n} . If $\mu =\frac{1}{3-\cos\frac{2\pi }{n}}$ , we have that $\mu g_{1}+\mu g_{2}+\left(1-2\mu \right)g_{3}=0_{n\times 1}$ , where $0_{n\times 1}$ is the n×1 zero matrix. The result now follows from [18] (p. 12) and the fact that a convex combination of subgradients of *f* at $\left(\gamma_{0},\ldots ,\gamma_{0}\right)$ is also a subgradient of *f* at $\left(\gamma_{0},\ldots ,\gamma_{0}\right)$ .

Case 2: Assume that *n* is odd. Then,

(A38)
$${∥\overset{\circ }{W}\left(\gamma_{0}\right)-P_{n}∥}_{2}=\;a_{2}\;=\;-a_{\frac{n+1}{2}}\;=\;-a_{\frac{n+3}{2}}\;=\;a_{n}=\frac{\cos\frac{2\pi }{n}+\cos\frac{\pi }{n}}{2-\cos\frac{2\pi }{n}+\cos\frac{\pi }{n}}\in \;\left(0,1\right).$$

Therefore, $y_{2}=\frac{\sqrt{2}}{2}\left(v_{n}+v_{2}\right)$ and $y_{n}=\frac{\sqrt{2}}{2\sqrt{-1}}\left(v_{n}-v_{2}\right)$ are unit eigenvectors of $\overset{\circ }{W}\left(\gamma_{0}\right)$ associated with $a_{2}=a_{n}$ , and $y_{\frac{n+1}{2}}=\frac{\sqrt{2}}{2}\left(v_{\frac{n+1}{2}}+v_{\frac{n+3}{2}}\right)$ and $y_{\frac{n+3}{2}}=\frac{\sqrt{2}}{2\sqrt{-1}}\left(v_{\frac{n+1}{2}}-v_{\frac{n+3}{2}}\right)$ are unit eigenvectors of $\overset{\circ }{W}\left(\gamma_{0}\right)$ associated with $a_{\frac{n+1}{2}}=a_{\frac{n+3}{2}}$ . As:

(A39)
$$\begin{array}{cc} {\left[y_{2}\right]}_{j,1} & =\sqrt{\frac{2}{n}}\cos\frac{2\pi (j-1)}{n}, \end{array}$$

(A40)
$$\begin{array}{cc} {\left[y_{n}\right]}_{j,1} & =\sqrt{\frac{2}{n}}\sin\frac{2\pi (j-1)}{n}, \end{array}$$

(A41)
$$\begin{array}{cc} {\left[y_{\frac{n+1}{2}}\right]}_{j,1} & =\sqrt{\frac{2}{n}}{\left(-1\right)}^{j-1}\cos\frac{\pi (j-1)}{n}, \end{array}$$

(A42)
$$\begin{array}{cc} {\left[y_{\frac{n+3}{2}}\right]}_{j,1} & =\sqrt{\frac{2}{n}}{\left(-1\right)}^{j-1}\sin\frac{\pi (j-1)}{n} \end{array}$$

for all j∈{1,…,n} , from [4] (Theorem 1), we obtain four subgradients of *f* at $\left(\gamma_{0},\ldots ,\gamma_{0}\right)\in R^{n}$ , namely $g_{1}$ , $g_{2}$ , $g_{3}$ and $g_{4}$ given by:

(A43)
$$\begin{array}{cc} {\left[g_{1}\right]}_{j,1} & =-\frac{2}{n}{\left(\cos\frac{2\pi (j-1)}{n}-\cos\frac{2\pi j}{n}\right)}^{2}, \end{array}$$

(A44)
$$\begin{array}{cc} {\left[g_{2}\right]}_{j,1} & =-\frac{2}{n}{\left(\sin\frac{2\pi (j-1)}{n}-\sin\frac{2\pi j}{n}\right)}^{2}, \end{array}$$

(A45)
$$\begin{array}{cc} {\left[g_{3}\right]}_{j,1} & =\frac{2}{n}{\left(\cos\frac{\pi (j-1)}{n}+\cos\frac{\pi j}{n}\right)}^{2}, \end{array}$$

(A46)
$$\begin{array}{cc} {\left[g_{4}\right]}_{j,1} & =\frac{2}{n}{\left(\sin\frac{\pi (j-1)}{n}+\sin\frac{\pi j}{n}\right)}^{2} \end{array}$$

for all j∈{1,…,n} . If $\mu =\frac{1}{2}\frac{1+\cos\frac{\pi }{n}}{2-\cos\frac{2\pi }{n}+\cos\frac{\pi }{n}}$ , we have that $\mu g_{1}+\mu g_{2}+\left(\frac{1}{2}-\mu \right)g_{3}+\left(\frac{1}{2}-\mu \right)g_{4}=0_{n\times 1}$ . The result now follows from [18] (p. 12) and the fact that a convex combination of subgradients of *f* at $\left(\gamma_{0},\ldots ,\gamma_{0}\right)$ is also a subgradient of *f* at $\left(\gamma_{0},\ldots ,\gamma_{0}\right)$ .

Since $∥\overset{\circ }{W}\left(\gamma_{0}\right)-P_{n}{∥}_{2}<1$ , applying [2] (Theorem 1) and Theorem A1, Theorem 1 holds.

## Appendix C. Proof of Theorem 2

From [2] (Theorem 1), Theorem A1, (A31) and (A38), we obtain (8).

To finish the proof, we only need to show (9) and (7).

We begin by proving (9). Applying Taylor’s theorem (see, e.g., [19] (p. 113)), there exist two bounded functions $f,g:\left(\left.0,\frac{\pi }{2}\right]\right.\to R$ such that:

(A47)
$$-\log\frac{1+\cos x}{3-\cos x}=\frac{x^{2}}{2}+f\left(x\right)x^{3}$$

and:

(A48)
$$-\log\frac{\cos\frac{x}{2}+\cos x}{2+\cos\frac{x}{2}-\cos x}=\frac{x^{2}}{2}+g\left(x\right)x^{3}$$

for all $x\in \left(\left.0,\frac{\pi }{2}\right]\right.$ . Therefore, from (A31) and (A38), we have:

(A49)
$$\frac{\log\epsilon^{-1}}{-\log∥\overset{\circ }{W}\left(\gamma_{0}\right)-P_{n}{∥}_{2}}=\frac{\log\epsilon^{-1}}{\frac{{\left(\frac{2\pi }{n}\right)}^{2}}{2}+y_{n}{\left(\frac{2\pi }{n}\right)}^{3}},$$

where ${\left\{y_{n}\right\}}_{n\geq 4}$ is the bounded sequence of real numbers given by:

(A50)
$${\left\{y_{n}\right\}}_{n\geq 4}=\left\{\begin{array}{cc} f\left(\frac{2\pi }{n}\right)\; & \mathrm{if}n\mathrm{is}\mathrm{even},\\ g\left(\frac{2\pi }{n}\right) & \mathrm{if}n\mathrm{is}\mathrm{odd}. \end{array}\right.$$

Thus,

(A51)
$$\lim_{n\to \infty }\frac{\log\epsilon^{-1}}{-n^{2}\log{∥\overset{\circ }{W}\left(\gamma_{0}\right)-P_{n}∥}_{2}}=\lim_{n\to \infty }\frac{\log\epsilon^{-1}}{2\pi^{2}+y_{n}\frac{8\pi^{3}}{n}}=\frac{\log\epsilon^{-1}}{2\pi^{2}}.$$

Hence, as a≤⌈a⌉<a+1 , with a∈R , applying Theorem A1, we obtain:

(A52)
$$\begin{array}{cc} \frac{\log\epsilon^{-1}}{2\pi^{2}} & =\lim_{n\to \infty }\frac{\log\epsilon^{-1}}{-n^{2}\log{∥\overset{\circ }{W}\left(\gamma_{0}\right)-P_{n}∥}_{2}}\leq \lim_{n\to \infty }\frac{\tau (\epsilon ,\overset{\circ }{W}\left(\gamma_{0}\right))}{n^{2}} \end{array}$$

(A53)
$$\begin{array}{c} \leq \lim_{n\to \infty }\frac{\log\epsilon^{-1}}{-n^{2}\log{∥\overset{\circ }{W}\left(\gamma_{0}\right)-P_{n}∥}_{2}}+\lim_{n\to \infty }\frac{1}{n^{2}}=\frac{\log\epsilon^{-1}}{2\pi^{2}}, \end{array}$$

and consequently, (9) holds.

Finally, we prove (7). If $\delta \in \left(0,\frac{\log\epsilon^{-1}}{2\pi^{2}}\right)$ , then there exists $n_{0}\in N$ , with $n_{0}>3$ , such that:

(A54)
$$\left|\frac{\tau (\epsilon ,\overset{\circ }{W}\left(\gamma_{0}\right))}{n^{2}}-\frac{\log\epsilon^{-1}}{2\pi^{2}}\right|<\delta \;\forall n\geq n_{0}.$$

Thus, if $n\geq n_{0}$ , then:

(A55)
$$-\delta <\frac{\tau (\epsilon ,\overset{\circ }{W}\left(\gamma_{0}\right))}{n^{2}}-\frac{\log\epsilon^{-1}}{2\pi^{2}}<\delta ,$$

or equivalently,

(A56)
$$\left(\frac{1}{2\pi^{2}}-\frac{\delta }{\log\epsilon^{-1}}\right)n^{2}\log\epsilon^{-1}<\tau \left(\epsilon ,\overset{\circ }{W}\left(\gamma_{0}\right)\right)<\left(\frac{1}{2\pi^{2}}+\frac{\delta }{\log\epsilon^{-1}}\right)n^{2}\log\epsilon^{-1}.$$

## Appendix D. Proof of Theorem 3

We denote with $\overset{⊠}{W}$ the set of all the rc×rc real symmetric matrices such that:

(A57)
$${\overset{⊠}{W}}_{r,c}=\left\{B\in R^{rc\times rc},\;B=B^{⊤},\;BP_{n}=P_{n},\;{\left[B\right]}_{j,k}=0\mathrm{if}j\neq k\mathrm{and}{\left[\overset{⊠}{A}\right]}_{j,k}=0\right\},$$

where $\overset{⊠}{A}$ is the adjacency matrix of a grid of *r* rows and *c* columns. Consider the bijection $B:\;R^{q}\mapsto {\overset{⊠}{W}}_{r,c}$ defined in [4] (Equation (8)), where q=4rc−3c−3r+2 (i.e., *q* is the number of edges when the network is viewed as an undirected graph).

We define the function $f:\;R^{q}\mapsto \left[0,\infty \right)$ as $f\left(w_{1},\ldots ,w_{q}\right):={∥B\left(w_{1},\ldots ,w_{q}\right)-P_{n}∥}_{2}$ . We next prove that:

(A58)
$${∥{\overset{⊠}{W}}_{r,c}\left(\frac{1}{2}\right)-P_{n}∥}_{2}\leq {∥B\left(w_{1},\ldots ,w_{q}\right)-P_{n}∥}_{2}\;\forall w_{1},\ldots ,w_{q}\in R.$$

Without loss of generality, we can assume that r≥c . We first show that ${\overset{⊠}{W}}_{r,c}\left(\frac{1}{2}\right)\in {\overset{⊠}{W}}_{r,c}$ :

(A59)
$$\left(\;{\tilde{W}}_{r}\left(\frac{1}{2}\right)⊗{\tilde{W}}_{c}\left(\frac{1}{2}\right)\;\right)\;{\;}^{⊤}\;\;\;=\;\;\left(\;{\tilde{W}}_{r}\left(\frac{1}{2}\right)\;\right)\;{\;}^{⊤}\;\;⊗\;\left(\;{\tilde{W}}_{c}\left(\frac{1}{2}\right)\;\right)\;{\;}^{⊤}\;\;={\tilde{W}}_{r}\left(\frac{1}{2}\right)⊗{\tilde{W}}_{c}\left(\frac{1}{2}\right),$$

and:

(A60)
$$\begin{array}{cc} \left({\tilde{W}}_{r}\left(\frac{1}{2}\right)⊗{\tilde{W}}_{c}\left(\frac{1}{2}\right)\right)P_{n} & \;=\;\left({\tilde{W}}_{r}\left(\frac{1}{2}\right)⊗{\tilde{W}}_{c}\left(\frac{1}{2}\right)\right)\;\left(\sqrt{n}P_{r}⊗\sqrt{n}P_{c}\right) \end{array}$$

(A61)
$$\begin{array}{c} =\;\;n\left(\left({\tilde{W}}_{r}\left(\frac{1}{2}\right)P_{r}\right)\;⊗\;\left({\tilde{W}}_{c}\left(\frac{1}{2}\right)P_{c}\right)\right) \end{array}$$

(A62)
$$\begin{array}{c} =n\left(P_{r}⊗P_{c}\right)=\sqrt{n}P_{r}⊗\sqrt{n}P_{c}=P_{n}. \end{array}$$

The eigenvalues of ${\tilde{W}}_{n}\left(\alpha \right)$ are (see, e.g., [20]):

(A63)
$$a_{j}:=1-2\alpha +2\alpha \left(\cos\frac{\pi (j-1)}{n}\right),\;j\in \left\{1,\ldots ,n\right\}$$

and therefore, the eigenvalues of ${\tilde{W}}_{n}\left(\frac{1}{2}\right)$ are given by $a_{j}\left(n\right):=\cos\frac{(j-1)\pi }{n}$ with j∈{1,…,n} . Their associated orthonormal eigenvectors are given by ${\left[v_{1}\left(n\right)\right]}_{k,1}=\frac{1}{\sqrt{n}}$ and ${\left[v_{j}\left(n\right)\right]}_{k,1}=\sqrt{\frac{2}{n}}\cos\frac{(2k-1)(j-1)\pi }{2n}$ with j∈{2,…,n} , k∈{1,…,n} (see, e.g., [20]). Consequently, the eigenvalues of ${\tilde{W}}_{r}\left(\frac{1}{2}\right)⊗{\tilde{W}}_{c}\left(\frac{1}{2}\right)$ are $a_{j}\left(r\right)a_{k}\left(c\right)$ and associated orthonormal eigenvectors are $v_{j}\left(r\right)⊗v_{k}\left(c\right)$ with j∈{1,…,r} and k∈{1,…,c} .

From [4] (Lemma 1),

(A64)
$${∥{\overset{⊠}{W}}_{r,c}\left(\frac{1}{2}\right)-P_{n}∥}_{2}=a_{2}\left(r\right)a_{1}\left(c\right)=-a_{r}\left(r\right)a_{1}\left(c\right)=\cos\frac{\pi }{r}\in \left(0,1\right).$$

Then, $y_{1}=v_{2}\left(r\right)⊗v_{1}\left(c\right)$ and $y_{2}=v_{r}\left(r\right)⊗v_{1}\left(c\right)$ are unit eigenvectors of ${\overset{⊠}{W}}_{r,c}\left(\frac{1}{2}\right)$ associated with $a_{2}\left(r\right)a_{1}\left(c\right)$ and $a_{r}\left(r\right)a_{1}\left(c\right)$ , respectively, and their entries are given by:

(A65)
$$\begin{array}{cc} {\left[y_{1}\right]}_{c(j-1)+k,1} & =\sqrt{\frac{2}{rc}}\cos\frac{(2j-1)\pi }{2r}, \end{array}$$

(A66)
$$\begin{array}{cc} y_{2}{]}_{c(j-1)+k,1} & ={\left(-1\right)}^{j-1}\sqrt{\frac{2}{rc}}\sin\frac{(2j-1)\pi }{2r}, \end{array}$$

for all j∈{1,…,r} and k∈{1,…,c} .

Let ε be the set of edges of the grid. An edge e={j,k} connects the sensors $v_{j},v_{k}$ , and we enumerate the edges such that $e_{l}=\left\{j_{l},k_{l}\right\}$ for all l∈{1,…,q} . We consider that the edges of the grid are sorted as follows: $\varepsilon_{H}$ , $\varepsilon_{V}$ , $\varepsilon_{\mathrm{NW}}$ and $\varepsilon_{\mathrm{NE}}$ are the set of horizontal, vertical, northwest-southeast diagonal and northeast-southwest diagonal edges, respectively. Moreover, if $e_{l_{1}}=\left\{j_{l_{1}},k_{l_{1}}\right\},e_{l_{2}}=\left\{j_{l_{2}},k_{l_{2}}\right\}\in \varepsilon_{h}$ with h∈{H,V,NW,NE} and $\min\left\{j_{l_{1}},k_{l_{1}}\right\}<\min\left\{j_{l_{2}},k_{l_{2}}\right\}$ , then the edge $e_{l_{1}}$ precedes the edge $e_{l_{2}}$ in $\varepsilon_{h}$ .

From [4] (Theorem 1) , we obtain two subgradients of *f*, $g_{1}$ and $g_{2}$ given by:

(A67)
$$\begin{array}{cc} g_{1} & ={\left(g_{1}^{\left(H\right)}|g_{1}^{\left(V\right)}\left|g_{1}^{\left(\mathrm{NW}\right)}\right|g_{1}^{\left(\mathrm{NE}\right)}\right)}^{⊤}, \end{array}$$

(A68)
$$\begin{array}{cc} g_{2} & ={\left(g_{2}^{\left(H\right)}|g_{2}^{\left(V\right)}\left|g_{2}^{\left(\mathrm{NW}\right)}\right|g_{2}^{\left(\mathrm{NE}\right)}\right)}^{⊤}, \end{array}$$

where $g_{1}^{\left(H\right)}=g_{2}^{\left(H\right)}=0_{1\times r(c-1)}$ ,

(A69)
$$\begin{array}{cc} {\left[g_{1}^{\left(V\right)}\right]}_{1,c(j-1)+k} & =-\frac{8}{rc}\sin^{2}\left(\frac{\pi }{2r}\right)\sin^{2}\left(\frac{j\pi }{r}\right), \end{array}$$

(A70)
$$\begin{array}{cc} {\left[g_{2}^{\left(V\right)}\right]}_{1,c(j-1)+k} & =\frac{8}{rc}\cos^{2}\left(\frac{\pi }{2r}\right)\sin^{2}\left(\frac{j\pi }{r}\right), \end{array}$$

for all j∈{1,…,r−1},k∈{1,…,c} ,

(A71)
$$\begin{array}{cc} {\left[g_{1}^{\left(\mathrm{NW}\right)}\right]}_{1,(c-1)(j-1)+k} & =-\frac{8}{rc}\sin^{2}\left(\frac{\pi }{2r}\right)\sin^{2}\left(\frac{j\pi }{r}\right), \end{array}$$

(A72)
$$\begin{array}{cc} {\left[g_{2}^{\left(\mathrm{NW}\right)}\right]}_{1,(c-1)(j-1)+k} & =\frac{8}{rc}\cos^{2}\left(\frac{\pi }{2r}\right)\sin^{2}\left(\frac{j\pi }{r}\right), \end{array}$$

for all j∈{1,…,r−1},k∈{1,…,c−1} , and:

(A73)
$$\begin{array}{cc} {\left[g_{1}^{\left(\mathrm{NE}\right)}\right]}_{1,(c-1)(j-1)+k-1} & =-\frac{8}{rc}\sin^{2}\left(\frac{\pi }{2r}\right)\sin^{2}\left(\frac{j\pi }{r}\right), \end{array}$$

(A74)
$$\begin{array}{cc} {\left[g_{2}^{\left(\mathrm{NE}\right)}\right]}_{1,(c-1)(j-1)+k-1} & =\frac{8}{rc}\cos^{2}\left(\frac{\pi }{2r}\right)\sin^{2}\left(\frac{j\pi }{r}\right), \end{array}$$

for all j∈{1,…,r−1} , k∈{2,…,c} . If $\mu =\cos^{2}\left(\frac{\pi }{2r}\right)$ , we have that $\mu g_{1}+\left(1-\mu \right)g_{2}=0_{(4rc-3c-3r+2)\times 1}$ . The result now follows from [18] (p. 12) and the fact that a convex combination of subgradients of *f* at a certain point is also a subgradient of *f* at that point.

Since $∥{\overset{⊠}{W}}_{r,c}\left(\frac{1}{2}\right)-P_{n}{∥}_{2}<1$ , applying [2] (Theorem 1) and Theorem A1, Theorem 3 holds.

## Appendix E. Proof of Theorem 9

We begin by proving (51). The Laplacian matrix of a cycle with *n* sensors is:

(A75)
$$\begin{array}{cc} \overset{\circ }{L} & =\mathrm{diag}\left(1_{n}^{⊤}{\mathrm{circ}}_{n}\left(0,1,0,\ldots ,0,1\right)\right)-{\mathrm{circ}}_{n}\left(0,1,0,\ldots ,0,1\right) \end{array}$$

(A76)
$$\begin{array}{c} =\mathrm{diag}\left(2,2,\ldots ,2\right)-{\mathrm{circ}}_{n}\left(0,1,0,\ldots ,0,1\right)={\mathrm{circ}}_{n}\left(2,-1,0,\ldots ,0,-1\right). \end{array}$$

From [21] (Equation (3.4a)), the eigenvalues of $\overset{\circ }{L}$ are given by $\left\{2\left(1-\cos\frac{2\pi (j-1)}{n}\right):\;j\in \left\{1,\ldots ,n\right\}\right\}$ and, consequently, $\lambda_{n-1}\left(\overset{\circ }{L}\right)=2\left(1-\cos\frac{2\pi }{n}\right)$ . From [7] (Corollary 1), we have:

(A77)
$${\overset{\circ }{\varphi }}_{0}=\frac{n-\lambda_{n-1}\left(\overset{\circ }{L}\right)}{2n-\lambda_{n-1}\left(\overset{\circ }{L}\right)}=1-\frac{n}{2\left(n+\cos\frac{2\pi }{n}-1\right)},$$

and therefore, (51) holds. The entries of the expectation of ${\overset{\circ }{W}}_{B}\left(0\right)$ are given by:

(A78)
$${\left[E\left({\overset{\circ }{W}}_{B}\left(0\right)\right)\right]}_{j,k}=\left\{\begin{array}{cc} \frac{1}{n}\left(1-{\overset{\circ }{\varphi }}_{0}\right) & \mathrm{if}j-k\in \{-1,1\},\\ \frac{1}{n}\left(1-{\overset{\circ }{\varphi }}_{0}\right) & \mathrm{if}j-k\in \{1-n,n-1\},\\ \frac{1}{n}\left(2{\overset{\circ }{\varphi }}_{0}+n-2\right) & \mathrm{if}j=k,\\ 0 & \mathrm{otherwise}, \end{array}\right.$$

for all j,k∈{1,…,n} . Thus, $E\left({\overset{\circ }{W}}_{B}\left(0\right)\right)={\overset{\circ }{W}}_{n}\left(\frac{1-{\overset{\circ }{\varphi }}_{0}}{n}\right)$ . Therefore, combining (A29) and (A30) yields:

(A79)
$${∥{\overset{\circ }{W}}_{n}\left(\frac{1-{\overset{\circ }{\varphi }}_{0}}{n}\right)-P_{n}∥}_{2}=\frac{n+2\cos\frac{2\pi }{n}-2}{n+\cos\frac{2\pi }{n}-1}.$$

As:

(A80)
$${\overset{\circ }{\varphi }}_{0}=\frac{n-\lambda_{n-1}\left(\overset{\circ }{L}\right)}{2n-\lambda_{n-1}\left(\overset{\circ }{L}\right)}=1-\frac{n}{2n-\lambda_{n-1}\left(\overset{\circ }{L}\right)}$$

we get:

(A81)
$$\lambda_{n-1}\left(\overset{\circ }{L}\right)=n\left(2-\frac{1}{1-{\overset{\circ }{\varphi }}_{0}}\right)=n\frac{1-2{\overset{\circ }{\varphi }}_{0}}{1-{\overset{\circ }{\varphi }}_{0}}$$

and consequently,

(A82)
$$\begin{array}{cc} & 1-\frac{2{\overset{\circ }{\varphi }}_{0}\left(1-{\overset{\circ }{\varphi }}_{0}\right)}{n}\lambda_{n-1}\left(\overset{\circ }{L}\right)-\frac{{\left(1-{\overset{\circ }{\varphi }}_{0}\right)}^{2}}{n^{2}}{\left(\lambda_{n-1}\left(\overset{\circ }{L}\right)\right)}^{2}=1-2{\overset{\circ }{\varphi }}_{0}\left(1-2{\overset{\circ }{\varphi }}_{0}\right)-{\left(1-2{\overset{\circ }{\varphi }}_{0}\right)}^{2} \end{array}$$

(A83)
$$\begin{array}{c} =1-2{\overset{\circ }{\varphi }}_{0}+4{\overset{\circ }{\varphi }}_{0}^{2}-1+4{\overset{\circ }{\varphi }}_{0}-4{\overset{\circ }{\varphi }}_{0}^{2}=2{\overset{\circ }{\varphi }}_{0}=2-\frac{n}{n+\cos\frac{2\pi }{n}-1}=\frac{n+2\cos\frac{2\pi }{n}-2}{n+\cos\frac{2\pi }{n}-1}. \end{array}$$

Now, applying (A79), (A82) and [7] (Equations (28) and (46)), we obtain (52). The rest of the proof runs as the proof of Theorem 2.

## Appendix F. Proof of Theorem 10

We begin by proving (56). The Laplacian matrix of a path with *n* sensors is:

(A84)
$${\left[\tilde{L}\right]}_{j,k}=\left\{\begin{array}{cc} -1 & \mathrm{if}\;j-k\in \{-1,1\},\\ 2 & \mathrm{if}\;j=k,j\neq 1\;\mathrm{and}\;j\neq n,\\ 1 & \mathrm{if}\;j=k,j\in \{1,n\},\\ 0 & \mathrm{otherwise}, \end{array}\right.$$

From [20], the eigenvalues of $\tilde{L}$ are given by $\left\{2\left(1-\cos\frac{\pi (j-1)}{n}\right):\;j\in \left\{1,\ldots ,n\right\}\right\}$ and, consequently, $\lambda_{n-1}\left(\tilde{L}\right)=2\left(1-\cos\frac{\pi }{n}\right)$ . From [7] (Corollary 1), we have:

(A85)
$${\tilde{\varphi }}_{0}=\frac{n-\lambda_{n-1}\left(\tilde{L}\right)}{2n-\lambda_{n-1}\left(\tilde{L}\right)}=1-\frac{n}{2\left(n+\cos\frac{\pi }{n}-1\right)},$$

and therefore, (56) holds. The entries of the expectation of ${\tilde{W}}_{B}\left(0\right)$ are given by:

(A86)
$${\left[E\left({\tilde{W}}_{B}\left(0\right)\right)\right]}_{j,k}=\left\{\begin{array}{cc} \frac{1}{n}\left(1-{\tilde{\varphi }}_{0}\right) & \mathrm{if}\;j-k\in \{-1,1\},\\ \frac{1}{n}\left(2{\tilde{\varphi }}_{0}+n-2\right) & \mathrm{if}\;j=k,j\neq 1\;\mathrm{and}\;j\neq n,\\ \frac{1}{n}\left({\tilde{\varphi }}_{0}+n-1\right) & \mathrm{if}\;j=k,j\in \{1,n\},\\ 0 & \mathrm{otherwise}, \end{array}\right.$$

for all j,k∈{1,…,n} . Thus, $E\left({\tilde{W}}_{B}\left(0\right)\right)={\tilde{W}}_{n}\left(\frac{1-{\tilde{\varphi }}_{0}}{n}\right)$ . Therefore, combining (A63) and [4] (Lemma 1) yields:

(A87)
$${∥{\tilde{W}}_{n}\left(\frac{1-{\tilde{\varphi }}_{0}}{n}\right)-P_{n}∥}_{2}=\frac{n+2\cos\frac{\pi }{n}-2}{n+\cos\frac{\pi }{n}-1}.$$

As:

(A88)
$${\tilde{\varphi }}_{0}=\frac{n-\lambda_{n-1}\left(\tilde{L}\right)}{2n-\lambda_{n-1}\left(\tilde{L}\right)}=1-\frac{n}{2n-\lambda_{n-1}\left(\tilde{L}\right)}$$

we get:

(A89)
$$\lambda_{n-1}\left(\tilde{L}\right)=n\left(2-\frac{1}{1-{\tilde{\varphi }}_{0}}\right)=n\frac{1-2{\tilde{\varphi }}_{0}}{1-{\tilde{\varphi }}_{0}}$$

and consequently,

(A90)
$$\begin{array}{cc} & 1-\frac{2{\tilde{\varphi }}_{0}\left(1-{\tilde{\varphi }}_{0}\right)}{n}\lambda_{n-1}\left(\tilde{L}\right)-\frac{{\left(1-{\tilde{\varphi }}_{0}\right)}^{2}}{n^{2}}{\left(\lambda_{n-1}\left(\tilde{L}\right)\right)}^{2}=1-2{\tilde{\varphi }}_{0}\left(1-2{\tilde{\varphi }}_{0}\right)-{\left(1-2{\tilde{\varphi }}_{0}\right)}^{2} \end{array}$$

(A91)
$$\begin{array}{c} =1-2{\tilde{\varphi }}_{0}+4{\tilde{\varphi }}_{0}^{2}-1+4{\tilde{\varphi }}_{0}-4{\tilde{\varphi }}_{0}^{2}=2{\tilde{\varphi }}_{0}=2-\frac{n}{n+\cos\frac{2\pi }{n}-1}=\frac{n+2\cos\frac{\pi }{n}-2}{n+\cos\frac{\pi }{n}-1}. \end{array}$$

Now, applying (A87), (A90) and [7] (Equations (28) and (46)), we obtain (57). The rest of the proof runs as the proof of Theorem 2.
